# New species of *Labiobaetis* Novikova & Kluge from Southeast Asia and New Guinea (Ephemeroptera, Baetidae)

**DOI:** 10.3897/zookeys.1067.72251

**Published:** 2021-11-01

**Authors:** Thomas Kaltenbach, Jean-Luc Gattolliat

**Affiliations:** 1 Museum of Zoology, Palais de Rumine, Place Riponne 6, CH-1005 Lausanne, Switzerland Museum of Zoology Lausanne Switzerland; 2 University of Lausanne (UNIL), Department of Ecology and Evolution, CH-1015 Lausanne, Switzerland University of Lausanne (UNIL) Lausanne Switzerland

**Keywords:** Biogeography, Borneo, COI, integrated taxonomy, morphology, Sulawesi

## Abstract

Material collected between 2006 and 2016 in Borneo, Sulawesi, and New Guinea further increased our knowledge of *Labiobaetis* Novikova & Kluge in these regions. Five species were previously reported from Borneo, two from Sulawesi, and 33 from New Guinea. Six new species have been identified using a combination of morphology and genetic distance (COI, Kimura 2-parameter), one species from Borneo (Brunei), one from Sulawesi, and four from New Guinea. They are described and illustrated based on their larvae and keys to the species of the relevant groups are provided. Additionally, new reports, a complementary description, and the COI sequence for *L.dendrisetis* Kaltenbach & Gattolliat are presented. The distribution of *Labiobaetis* in the Wallacea region is discussed based on the new findings. The total number of *Labiobaetis* species worldwide is augmented to 153.

## Introduction

The family Baetidae has the highest species diversity among mayflies, comprising ca. 1,100 species in 114 genera (updated from Sartori and Brittain 2015; Jacobus et al. 2019; Cruz et al. 2020), which is approximately one third of all mayfly species worldwide. They have a cosmopolitan distribution except New Zealand (Gattolliat and Nieto 2009). Investigations of the molecular phylogeny of the Order Ephemeroptera revealed the relatively basal position of the family in Ephemeroptera phylogeny (Ogden and Whiting 2005; Ogden et al. 2009, 2019).

The genus *Labiobaetis* Novikova & Kluge, 1987 (Novikova and Kluge 1987) is one of the richest genera of mayflies with 147 previously described species (Barber-James et al. 2013; Kaltenbach et al. 2020 and citations therein, 2021; Kaltenbach and Gattolliat 2021a, b). The distribution of *Labiobaetis* is nearly worldwide, except for the Neotropical realm, New Zealand, New Caledonia and some remote islands. The history and concept of the genus *Labiobaetis* were recently summarised in detail (Shi and Tong 2014; Kaltenbach and Gattolliat 2018). Kluge and Novikova (2016) established a new tribe Labiobaetini including the genera *Labiobaetis* and *Pseudopannota* Waltz & McCafferty, 1987, based on a unique combination of imaginal and larval characters.

Kaltenbach and Gattolliat (2018) started to create groups of species inside *Labiobaetis* based on combinations of morphological characters and later added further groups from other regions (Kaltenbach and Gattolliat 2019; Kaltenbach et al. 2020). In total, 16 groups were characterised so far. These morphological groups are primarily a working tool but could also serve as a basis for future studies on the generic delimitation and phylogeny of this genus. The inclusion of nuclear gene sequences may prove that some are natural groups.

This contribution will focus on further new species of *Labiobaetis* from Borneo, Sulawesi and New Guinea with integrative taxonomy. In the past, five species were reported from Indonesia (*L.fulmeki* (Ulmer), *L.obscurum* (Ulmer), *L.necopinatum* (Müller-Liebenau), *L.ulmeri* (Müller-Liebenau) and *L.boettgeri* (Ulmer)). All were described from adults only and no species were previously known at the larval stage (Ulmer 1913, 1924, 1939; Müller-Liebenau 1981). The generic attribution of these species is still controversial as *Labiobaetis* remains difficult to delimit in the imaginal stage. Recently, a first comprehensive study on *Labiobaetis* in Indonesia was done, including the description of 18 new species based on larvae (Kaltenbach and Gattolliat 2019). The *Labiobaetis* fauna of Borneo, including Brunei, the Malaysian part and the Indonesian part of the island was studied by Kaltenbach and Gattolliat (2020), after a first contribution by Müller-Liebenau (1984a). From the megadiverse New Guinea, the first six *Labiobaetis* species were reported by Lugo-Ortiz et al. (1999) and a subsequent larger study was published by Kaltenbach and Gattolliat (2019), including the description of 26 new species.

Indonesia is an immense archipelago of more than 18.000 islands extending over a huge area from 95°E to 141°E and from 6°N to 11°S. It is one of the most biologically rich countries in the world. The high levels of species richness and endemism are mainly attributable to a complex geological history, that brought together two different biological realms (Oriental realm and Australasian realm), separated by a transitional region (Wallacea) (Kingston 2010; Hall 2010). The main islands are Sumatra, Java, Borneo (partly, Kalimantan Province), Sulawesi, and New Guinea (partly, provinces West Papua and Papua). Borneo, Sumatra, Java, and the Malay Peninsula form the Sundaland Biodiversity Hotspot (Quek 2010), influenced by a dynamic and highly complex geophysical history including changing climates, fluctuating sea levels, volcanism, and orogenic activity with subsequent erosion (Quek 2010). New Guinea, the second largest island after Greenland, is equally known for its megadiversity. It is a geological composite consisting of many separate terranes; the evolutionary history of the biota is linked to the accretion of these terranes to the Australian craton, and to the uplift, volcanism, and rifting that accompanied these tectonic events (Allison 2010). There is strong evidence that recent environmental change in the extremely structured central highlands of New Guinea with its ongoing formation of rich aquatic resources and remote valleys and mountain blocks has been the primary driver of diversification of aquatic insects in that area (Toussaint et al. 2013, 2014). Taking into account the extreme diversity in Southeast Asia and New Guinea, the rather poor collection activities in the past, with many still unexplored regions, and the obvious richness of *Labiobaetis* in this region, we have to expect many more species with further collections in the future.

## Materials and methods

Part of the material was collected during a series of university training practicals (see also Kaltenbach et al. 2021). The specimens from Brunei were collected in 2014 and 2016 by Kate Baker (University of Exeter, UK) during ecological studies in Brunei Darussalam in collaboration with Universiti Brunei Darussalam (Baker et al. 2016a, b, 2017a, b, 2019).

All specimens were preserved in 70%–96% ethanol. The dissection of larvae was done in Cellosolve (2-Ethoxyethanol) with subsequent mounting on slides with Euparal liquid, using an Olympus SZX7 stereomicroscope.

The DNA of part of the specimens was extracted using non-destructive methods allowing subsequent morphological analysis (see Vuataz et al. 2011 for details). We amplified a 658 bp fragment of the mitochondrial gene cytochrome oxidase subunit 1 (COI) using the primers LCO 1490 and HCO 2198 (Folmer et al. 1994; see Kaltenbach and Gattolliat 2020 for details). Sequencing was done with Sanger’s method (Sanger et al. 1977). The genetic variability between specimens was estimated using Kimura-2-parameter distances (K2P, Kimura 1980), calculated with the program MEGA 7 (Kumar et al. 2016, http://www.megasoftware.net).

The GenBank accession numbers are given in Table 1; the nomenclature of gene sequences follows Chakrabarty et al. (2013).

**Table 1.** Sequenced specimens: treated species and known species of group *claudiae*.

Species	Species group	Locality	Specimens catalog #	GenBank # (COI)	GenSeq Nomenclature
*L.catadupa* sp. nov.	*catadupa*	Brunei	GBIFCH00592439	MW868314	genseq-2 COI
*L.toraja* sp. nov.	*catadupa*	Sulawesi	GBIFCH00674627	MW868315	genseq-2 COI
			GBIFCH00674628	MW868316	genseq-2 COI
*L.academicus*	*claudiae*	Papua Province	GBIFCH00673069	MW041241	genseq-2 COI
			GBIFCH00673081	MW041242	genseq-2 COI
*L.centralensis*	*claudiae*	Papua New Guinea: Central Prov.	GBIFCH00465215	MH619495	genseq-1-COI
			GBIFCH00465216	MH619494	genseq-2 COI
*L.claudiae*	*claudiae*	Papua New Guinea: Madang Prov.	GBIFCH00508144	MH619479	genseq-1-COI
*L.hattam* sp. nov.	*claudiae*	Papua Barat	GBIFCH00763707	MW868311	genseq-2 COI
*L.stagnum*	*claudiae*	Papua Province	GBIFCH00465168	MH619491	genseq-2 COI
*L.werneri* sp. nov.	*claudiae*	Papua New Guinea: Gulf Prov.	GBIFCH00763699	MW868307	genseq-1-COI
		Papua New Guinea: Eastern Highlands	GBIFCH00763603	MW868309	genseq-2 COI
		Papua New Guinea: Morobe Prov.	GBIFCH00763700	MW868308	genseq-2 COI
*L.dendrisetis*	*dendrisetis*	Papua New Guinea: Central Prov.	GBIFCH00763706	MW868310	genseq-4 COI
*L.arfak* sp. nov.	*seramensis*	Papua Barat	GBIFCH00763714	MW868312	genseq-2 COI
			GBIFCH00763715	MW868313	genseq-2 COI

Drawings were made using an Olympus BX43 microscope. To facilitate the determination of species and the comparison of important structures, we partly used a combination of dorsal and ventral aspects in one drawing. Explanations are given in Kaltenbach et al. (2020: fig. 1).

**Figure 1.**
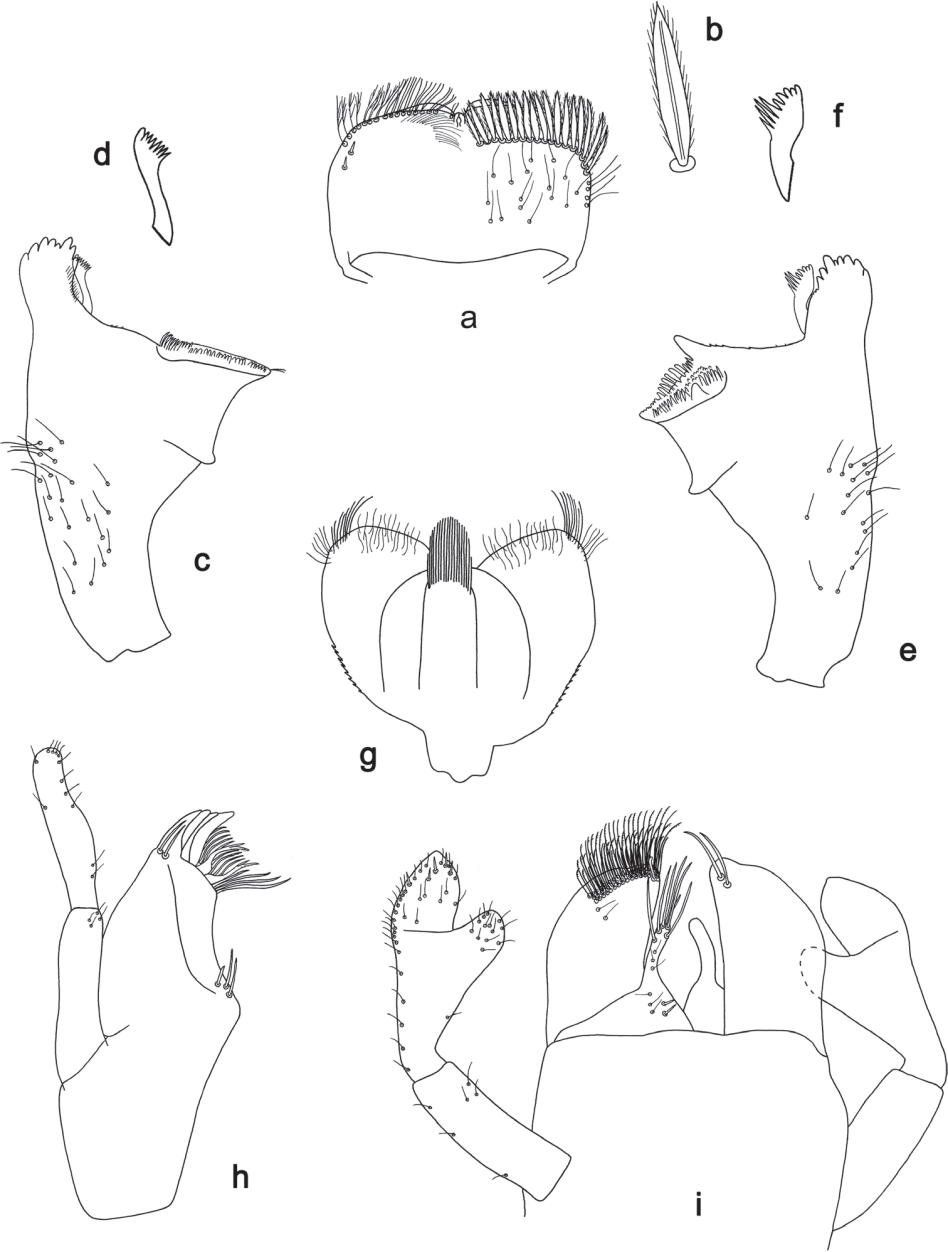
*Labiobaetiscatadupa* sp. nov., larva morphology: **a** labrum **b** seta from arc on dorsal surface of labrum **c** right mandible **d** right prostheca **e** left mandible **f** left prostheca **g** hypopharynx and superlingua **h** maxilla **i** labium.

Photographs of larvae were taken using a Canon EOS 6D camera and processed with the programs Adobe Photoshop Lightroom (http://www.adobe.com) and Helicon Focus version 5.3 (http://www.heliconsoft.com). Photographs were subsequently enhanced with Adobe Photoshop Elements 13.

The distribution maps were generated with the program SimpleMappr (https://simplemappr.net, Shorthouse 2010).

The dichotomous keys were elaborated with the support of the program DKey version 1.3.0 (http://drawwing.org/dkey, Tofilski 2018).

The terminology follows Hubbard (1995; legs orientation) and Kluge (2004; most terms, but the term gill/gills is used instead of tergalius/tergalii).

### Abbreviations

**MZB**Museum Zoologicum Bogoriense (Indonesia);

**MZL**Musée de Zoologie Lausanne (Switzerland);

**ZSM**Zoologische Staatssammlung München (Germany).

## Results

### List of *Labiobaetis* species treated in this paper

*catadupa* group (new group)

1. *L.catadupa* sp. nov.

2. *L.toraja* sp. nov.

*claudiae* group

3. *L.hattam* sp. nov.

4. *L.werneri* sp. nov.

dendrisetis group

5. *L.dendrisetis* Kaltenbach & Gattolliat, 2018

*seramensis* group

6. *L.arfak* sp. nov.

7. *L.onim* sp. nov.

### *Labiobaetiscatadupa* group of species (new group of species)

**Figure 2.**
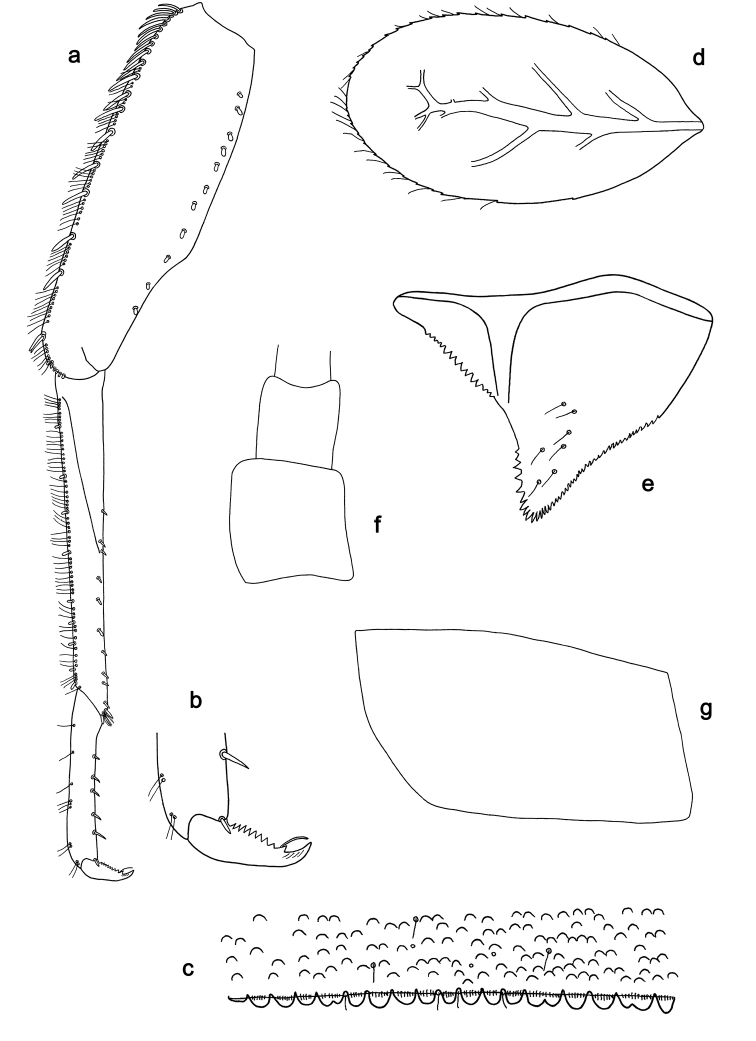
*Labiobaetiscatadupa* sp. nov., larva morphology: **a** foreleg **b** fore claw **c** tergum IV **d** gill IV **e** paraproct **f** base of antenna **g** metanotum.

**Figure 3.**
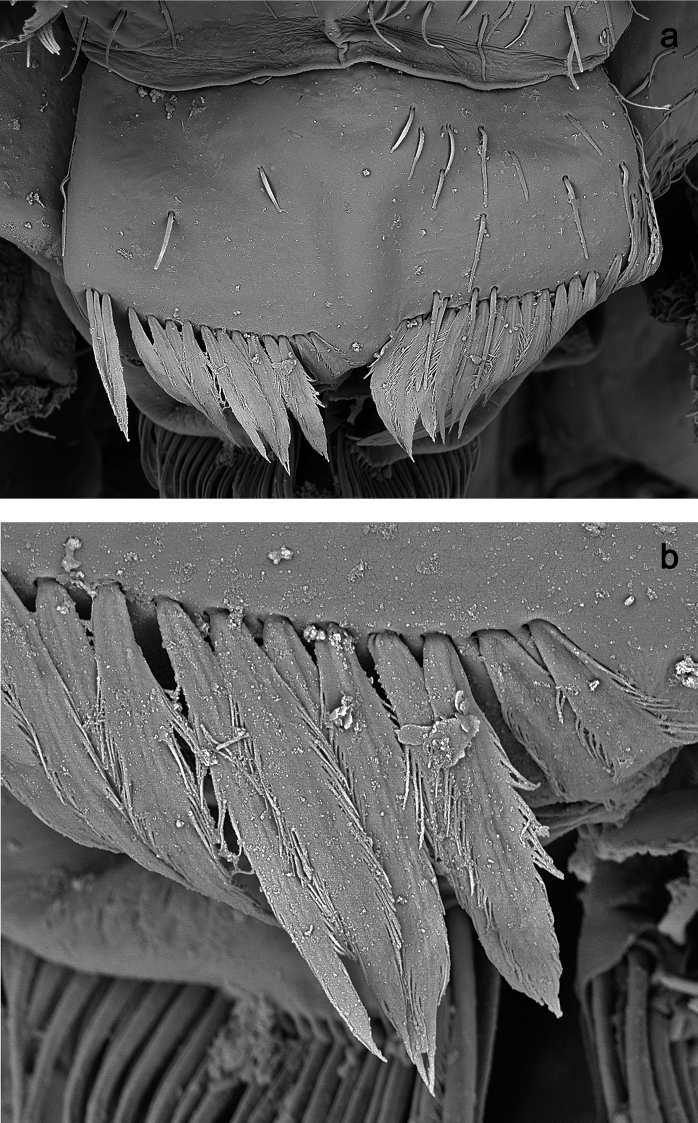
*Labiobaetiscatadupa* sp. nov., SEM pictures: **a** labrum **b** section of labrum with setae of dorsal, submarginal arc.

**Figure 4.**
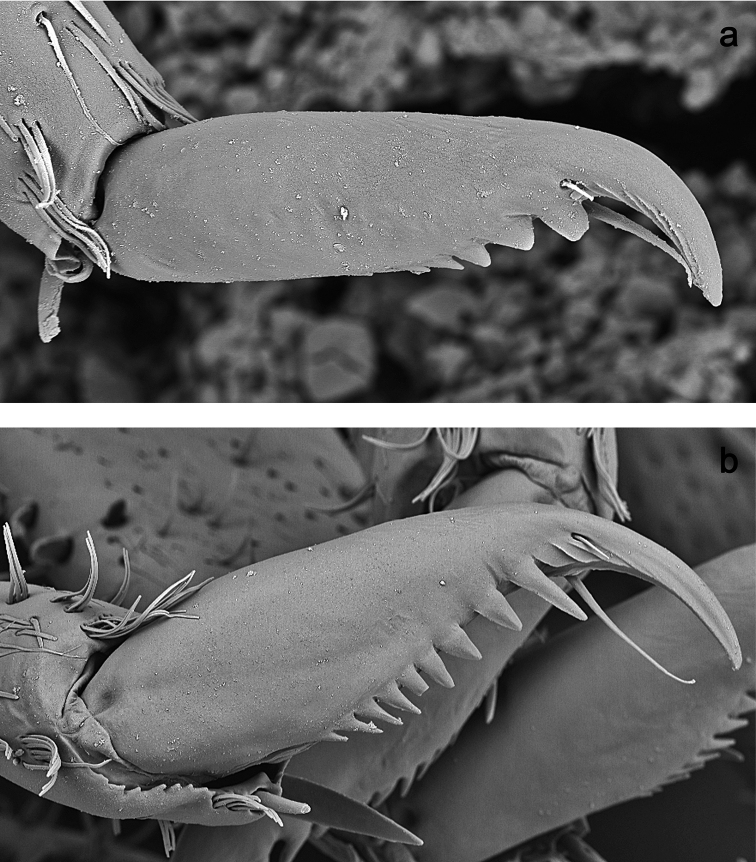
SEM pictures, claws: **a***Labiobaetiscatadupa* sp. nov. **b***Labiobaetistoraja* sp. nov.

The *catadupa* group can be recognised by the following combination of characters: A) dorsal surface of labrum with submarginal arc of feathered setae (Figs 1b, 6a, 3); B) labial palp segment II extended thumb-like, glossae much shorter than paraglossae (Figs 1i, 6h); C) claws with long subapical seta on posterior side and reduced subapical seta on anterior side (Figs 2b, 4a, b, 7d); D) hind protoptera absent; E) six pairs of gills (gill I absent).

The *L.catadupa* group is so far known from Borneo and Sulawesi, it includes the following species:

*Labiobaetiscatadupa* sp. nov.

*Labiobaetistoraja* sp. nov.

#### Labiobaetis catadupa sp. nov.

Taxon classificationAnimaliaEphemeropteraBaetidae

57A5A46F-A7E2-5597-BFF4-B9A6252B520A

http://zoobank.org/44D4E549-FDB5-41B8-B459-8EBB70BFB2E5

##### Type material.

***Holotype*.** Brunei • larva; Temburong District, Ulu Temburong National Park, Belalong River (near field station); 04°32'49"N, 115°09'30"E; 100 m; v. 2014; leg. K. Baker; on slide; GBIFCH00592448; MZL. ***Paratypes*.** Brunei • 17 larvae; Temburong District, Ulu Temburong National Park; 04°33'10"N, 115°09'20"E; v. 2014; leg. K. Baker; 2 on slides; GenBank MW868314; GBIFCH00592439, GBIFCH00592440; 15 in alcohol; GBIFCH00515571, GBIFCH00515573, GBIFCH00515598; MZL • 12 larvae; Temburong District, Ulu Temburong National Park; 04°32'42"N, 115°09'31"E; v. 2014; leg. K. Baker; 1 on slide; GBIFCH00592442; 11 in alcohol; GBIFCH00515597; MZL • 4 larvae; Temburong District, Ulu Temburong National Park; 04°32'23"N, 115°09'34"E; 25.–31.vii. 2016; leg. K. Baker; in alcohol; GBIFCH00515576; MZL • 9 larvae; Temburong District, Ulu Temburong National Park, Mata Ikan; 04°32'51"N, 115°09'25"E; 25.–31.vii. 2016; leg. K. Baker; 1 on slide; GBIFCH00592441; 8 in alcohol; GBIFCH00515582, GBIFCH00515583, GBIFCH00515589, GBIFCH00515590; MZL • 12 larvae; Temburong District, Ulu Temburong National Park; 04°33'39"N, 115°08'54"E; 25.–31.vii. 2016; leg. K. Baker; in alcohol; GBIFCH00515574, GBIFCH515575, GBIFCH515578; MZL • 5 larvae; Temburong District, Ulu Temburong National Park; 04°33'39"N, 115°08'51"E; 25.–31.vii. 2016; leg. K. Baker; 1 on slide; GBIFCH00515577; 4 in alcohol; GBIFCH00515599, GBIFCH00515572, GBIFCH00515621, GBIFCH00515600; MZL • 6 larvae; Temburong District, Ulu Temburong National Park; 04°33'10"N, 115°09'20"E; 25.–31.vii. 2016; leg. K. Baker; in alcohol; GBIFCH00515588, GBIFCH00515593; MZL. **Other material.** Brunei • 25 larvae; Temburong District, Ulu Temburong National Park; 04°32'42"N, 115°09'31"E; 25.–31.vii. 2016; leg. K. Baker; in alcohol; GBIFCH00515584, GBIFCH00515579, GBIFCH00515587, GBIFCH00515592; MZL • 5 larvae; Temburong District, Ulu Temburong National Park; 04°32'56"N, 115°09'27"E; 25.–31.vii. 2016; leg. K. Baker; in alcohol; GBIFCH00515580, GBIFCH00515586, GBIFCH00515591; MZL.

##### Diagnosis.

**Larva.** Following combination of characters: A) dorsal surface of labrum with submarginal arc of 17–19 long, feathered setae with broad middle part (Figs 1a, b, 3); B) labial palp segment II with extended thumb-like distomedial protuberance, segment III slightly pentagonal; glossae much shorter than paraglossae (Fig. 1i); C) left mandible without setae at apex of mola; D) fore femur length ca. 3× maximum width, dorsal margin with 17–19 curved, spine-like setae (Fig. 2a); E) claw with long subapical seta on posterior side and reduced subapical seta on anterior side (Figs 2b, 4a); F) hind protoptera absent; G) six pairs of gills (gill I absent); H) paraproct distally expanded, with ca. 40 stout, marginal spines.

##### Description.

**Larva** (Figs 1–3, 4a, 17a). Body length 2.6–6.0 mm. Cerci: ca. 2/3 of body length. Paracercus: ca. 1/5 of cerci length. Antenna: approx. twice as long as head length.

***Colouration*** (Fig. 17a). Head, thorax and abdomen dorsally and ventrally brown. Legs light brown, caudalii light brown.

***Antenna*** (Fig. 2f) with scape and pedicel sub cylindrical, without distolateral process at scape.

***Labrum*** (Fig. 1a, b). Rectangular, length 0.6× maximum width. Distal margin with medial emargination and a small process. Dorsally with medium, fine, simple setae scattered over surface; submarginal arc of setae composed of 17–19 long, feathered setae with broad middle part. Ventrally with marginal row of setae composed of anterolateral long, feathered setae and medial long, bifid setae; ventral surface with ca. two short, spine-like setae near lateral and anterolateral margin.

***Right mandible*** (Fig. 1c, d). Incisor and kinetodontium fused. Incisor with five denticles; kinetodontium with three denticles, inner margin of innermost denticle with a row of thin setae. Prostheca robust, apically denticulate. Margin between prostheca and mola slightly convex, with few minute denticles. Tuft of setae at apex of mola present.

***Left mandible*** (Fig. 1e, f). Incisor and kinetodontium fused. Incisor with five denticles; kinetodontium with three denticles. Prostheca robust, apically with small denticles and comb-shaped structure. Margin between prostheca and mola straight, with minute denticles. Subtriangular process long and slender, above level of area between prostheca and mola. Denticles of mola apically constricted. Tuft of setae at apex of mola absent.

Both mandibles with lateral margins slightly convex. Basal half with fine, simple setae scattered over dorsal surface.

***Hypopharynxandsuperlinguae*** (Fig. 1g). Lingua shorter than superlingua. Lingua approx. as long as broad; distal half laterally not expanded; medial tuft of stout setae well developed. Superlinguae distally rounded; lateral margins rounded; fine, long, simple setae along distal margin.

***Maxilla*** (Fig. 1h). Galea-lacinia ventrally with two simple, apical setae under canines. Inner dorsal row of setae with three denti-setae, distal denti-seta tooth-like, middle and proximal denti-setae slender, bifid and pectinate. Medially with one pectinate, spine-like seta and two or three medium, simple setae. Maxillary palp ca. 1.2× length of galea-lacinia; 2-segmented; palp segment II approx. as long as segment I; setae on maxillary palp fine, simple, scattered over surface of segments I and II; apex of last segment with slight excavation at inner distolateral margin.

***Labium*** (Fig. 1i). Glossa basally broad, narrowing toward apex; much shorter than paraglossa; inner margin with one spine-like seta; apex with two long and one medium, robust setae; outer margin with three spine-like setae; ventral surface with fine, simple, scattered setae. Paraglossa sub-rectangular, slightly curved inward; apex slightly concave; with three rows of long, robust, distally pectinate setae in apical area and two or three short, simple setae in anteromedial area; dorsally with two long, spine-like setae near inner margin. Labial palp with segment I 0.7× length of segments II and III combined. Segment I ventrally with short, fine, simple setae. Segment II with extended thumb-like, distomedial protuberance, bent upwards; distomedial protuberance 0.8× width of base of segment III; ventral surface with short, fine, simple setae; dorsally without spine-like setae near outer margin. Segment III slightly pentagonal; length 1.3× width; ventrally covered with short, spine-like, simple setae and short, fine, simple setae.

***Hind protoptera*** (Fig. 2g) absent.

***Foreleg*** (Fig. 2a, b). Ratio of foreleg segments 1.2:1.0:0.5:0.2. ***Femur*.** Length ca. 3× maximum width. Dorsal margin with a row of 17–19 curved, spine-like setae and a row of long, fine, simple setae; length of setae 0.24× maximum width of femur. Apex rounded, with a pair of spine-like setae and some short, stout setae. Stout, apically rounded setae scattered along ventral margin; femoral patch absent. ***Tibia*.** Dorsal margin with a row of short, spine-like setae and long, fine, simple setae. Ventral margin with a row of short, curved, spine-like setae, on apex a tuft of fine, simple setae. Patellotibial suture present on basal 1/2. ***Tarsus*.** Dorsal margin with a row of fine, simple setae. Ventral margin with a row of curved, spine-like setae. Claw with one row of eight or nine denticles; distally pointed; with ca. four stripes; with long, subapical seta on posterior side and reduced, subapical seta on anterior side.

***Terga*** (Fig. 2c). Surface with irregular rows of U-shaped scale bases and scattered fine, simple setae. Posterior margin of tergum IV with rounded spines, wider than long.

***Gills*** (Fig. 2d). Present on segments II–VII. Margin with small denticles intercalating fine simple setae. Tracheae extending from main trunk to inner and outer margins. Gill IV as long as length of segments V and 1/3 VI combined. Gill VII as long as length of segment VIII.

***Paraproct*** (Fig. 2e). Distally expanded, with ca. 40 stout, marginal spines. Surface scattered with fine, simple setae. Cercotractor with numerous small, marginal spines.

##### Etymology.

Based on the Latin word *catadupa*, meaning waterfall, with reference to the habitat of the species.

##### Distribution.

Brunei (Fig. 21b).

##### Biological aspects.

**Figure 5.**
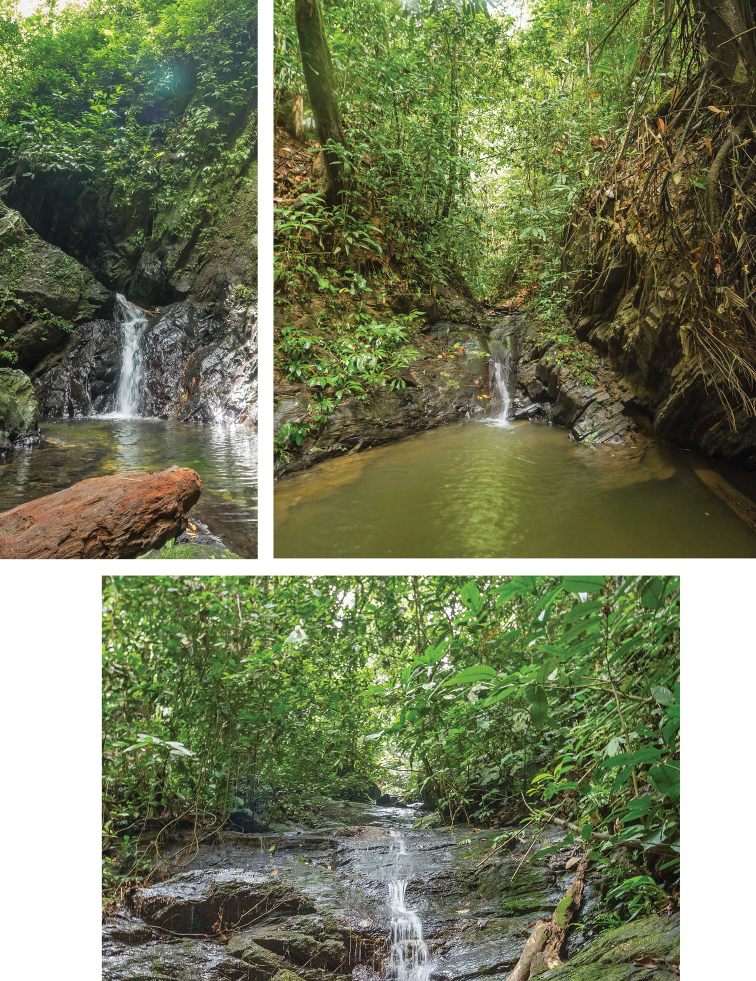
*Labiobaetiscatadupa* sp. nov., habitats in Brunei (photographs Kate Baker, University Exeter, UK).

The specimens were collected at an altitude of 150 m, mostly from waterfalls with slope angles of 16° to 50° and lengths between 5 m and 20 m (Fig. 5; Baker et al. 2017a, b). They were sampled on rock in fast flowing water and others it was a film of water with algae/moss (pers. comm. Kate Baker, University Exeter, Great Britain).

#### Labiobaetis toraja sp. nov.

Taxon classificationAnimaliaEphemeropteraBaetidae

7C205DA2-B480-5F52-B076-D81DDB8FD8F1

http://zoobank.org/03D69309-BC3E-4BB0-975A-84F99294AEF9

##### Type material.

***Holotype*.** Indonesia • larva; Sulawesi; Tengah, Lake Lore; 01°19'35"S, 120°18'40"E; 1600 m; 01.ix.2011; leg. Sumoked (SUL013); on slide; GBIFCH00592443; MZB. ***Paratypes*.** Indonesia • 9 larvae; same data as holotype; 2 on slides; GBIFCH00592444, GBIFCH00592446; 7 in alcohol; GenBank MW868315, MW868316; GBIFCH00674627, GBIFCH00674628, GBIFCH00515619, GBIFCH00515620, GBIFCH00515596; MZB, MZL.

##### Diagnosis.

**Figure 6.**
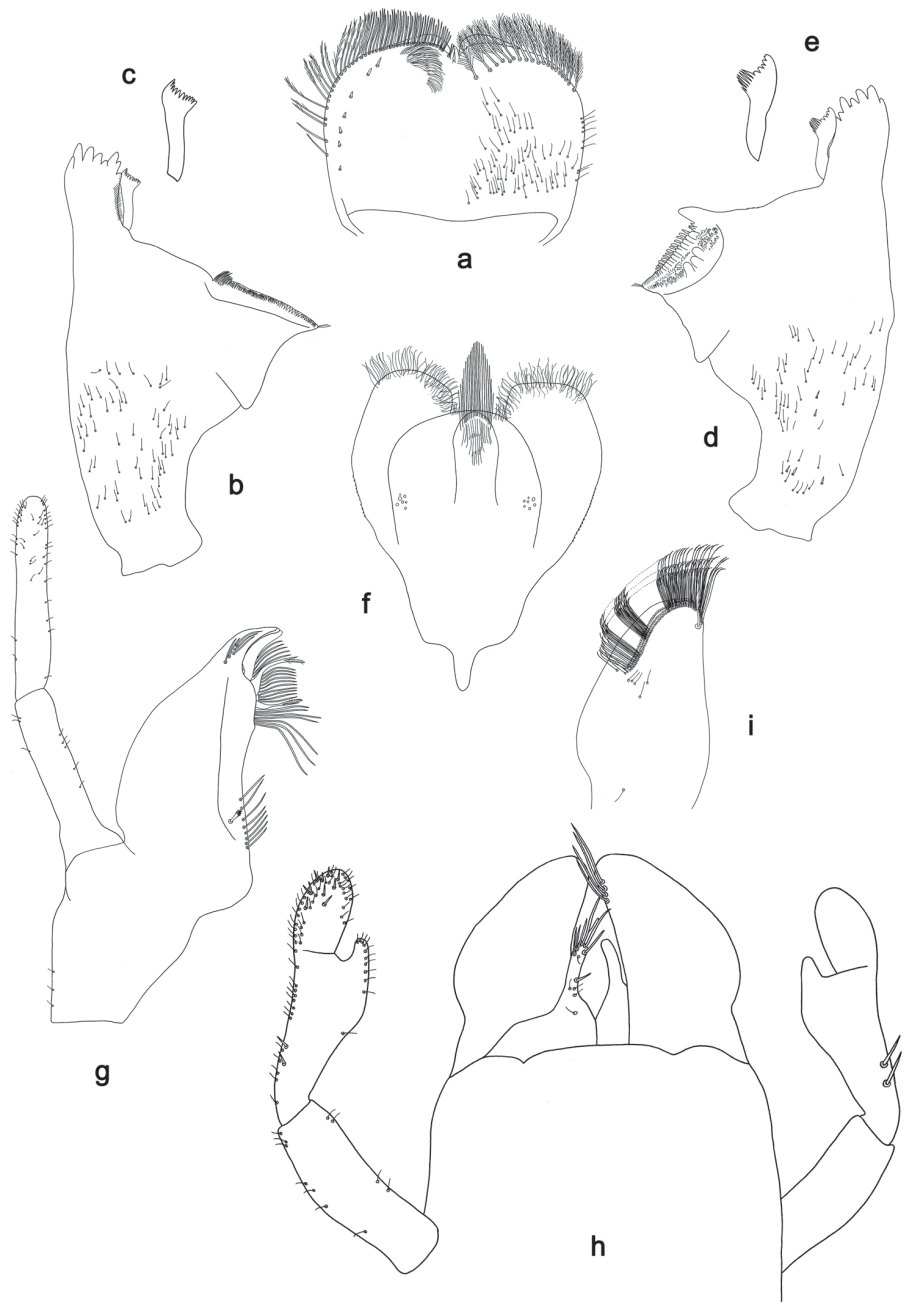
*Labiobaetistoraja* sp. nov., larva morphology: **a** labrum **b** right mandible **c** right prostheca **d** left mandible **e** left prostheca **f** hypopharynx and superlingua **g** maxilla **h** labium **i** apex of paraglossa.

**Figure 7.**
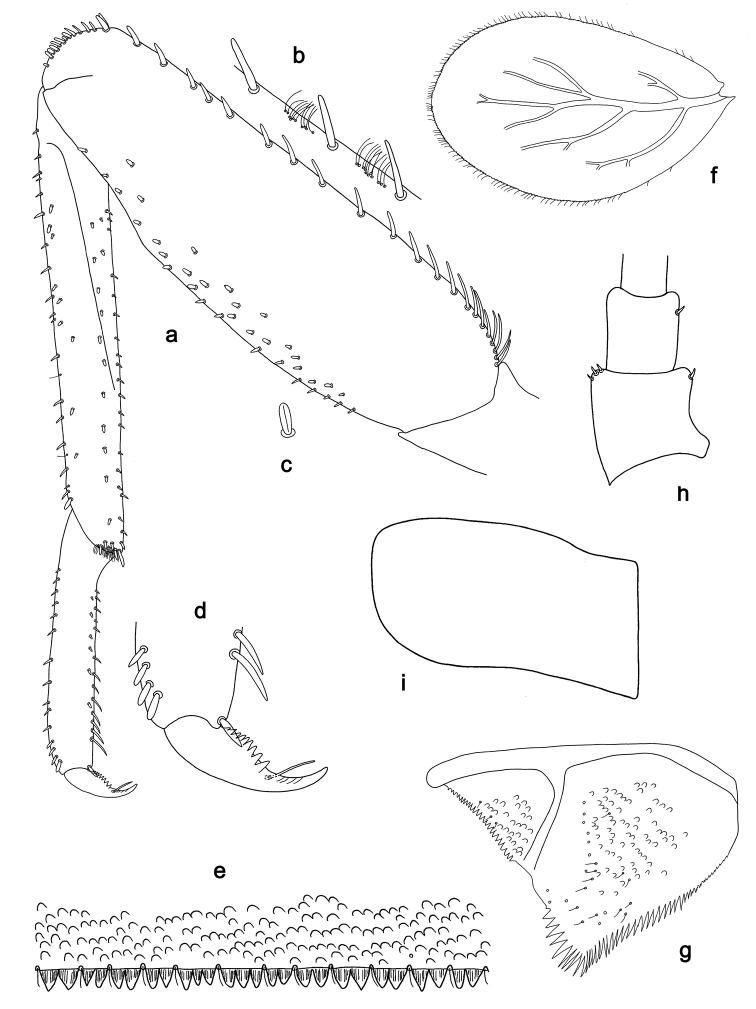
*Labiobaetistoraja* sp. nov., larva morphology: **a** foreleg **b** dorsal margin of femur **c** seta on ventral part of femur **d** fore claw **e** tergum IV **f** gill IV **g** paraproct **h** base of antenna **i** metanotum.

**Larva.** Following combination of characters: A) dorsal surface of labrum with submarginal arc of one plus 18–21 long, feathered setae (Figs 6a, 4b); B) labial palp segment II with hook-like distomedial protuberance, segment III oblong; glossae much shorter than paraglossae (Fig. 6h); C) left mandible with setae at apex of mola; D) fore femur length ca. 3× maximum width, dorsal margin with 18–25 curved, spine-like setae (Fig. 7a); E) claw with long subapical seta on posterior side and reduced subapical seta on anterior side (Figs 4b, 7d); F) hind protoptera absent; G) six pairs of gills (gill I absent); H) paraproct distally slightly expanded, with more than 40 stout, marginal spines.

##### Description.

**Larva** (Figs 4b, 6, 7, 17b, c). Body length 5.5–6.5 mm. Cerci broken. Paracercus: ca. 0.4× body length. Antenna: approx. twice as long as head length.

***Colouration*** (Fig. 17b, c). Head, thorax and abdomen dorsally brown, with pattern as in Fig. 17b. Head, thorax and abdomen ventrally light brown, abdominal segments VII–IX laterally darker (Fig. 17c). Legs light brown; femur with dorsomedial brown streak and brown sections apically and distoventrally; tibia basally and tarsus distally darker (Fig. 17c). Caudalii ecru.

***Antenna*** (Fig. 7h) with scape and pedicel sub cylindrical, without distolateral process at scape. Scape and pedicel with few stout setae.

***Labrum*** (Fig. 6a). Sub-rectangular, length 0.7× maximum width. Distal margin with medial emargination and a small process. Dorsally with medium, fine, simple setae scattered over surface; submarginal arc of setae composed of one plus 18–21 long, feathered setae. Ventrally with marginal row of setae composed of lateral and anterolateral long, feathered setae and medial long, bifid setae; ventral surface with ca. seven short, spine-like setae near lateral and anterolateral margin.

***Right mandible*** (Fig. 6b, c). Incisor and kinetodontium fused. Incisor with five denticles; kinetodontium with three denticles, inner margin of innermost denticle with a row of thin setae. Prostheca robust, apically denticulate. Margin between prostheca and mola straight. Tuft of setae at apex of mola present.

***Left mandible*** (Fig. 6d, e). Incisor and kinetodontium fused. Incisor with five denticles; kinetodontium with three denticles. Prostheca robust, apically with small denticles and comb-shaped structure. Margin between prostheca and mola straight, with few minute denticles. Subtriangular process long and slender, above level of area between prostheca and mola. Denticles of mola apically constricted. Tuft of setae at apex of mola present.

Both mandibles with lateral margins slightly convex. Basal half with fine, simple setae scattered over dorsal surface.

***Hypopharynxandsuperlinguae*** (Fig. 6f). Lingua shorter than superlingua. Lingua approx. as long as broad; distal half laterally not expanded; medial tuft of stout setae well developed and long. Superlinguae distally rounded; lateral margins rounded; fine, long, simple setae along distal margin.

***Maxilla*** (Fig. 6g). Galea-lacinia ventrally with five simple, apical setae under canines. Inner dorsal row of setae with three denti-setae, distal denti-seta tooth-like, middle and proximal denti-setae slender, bifid and pectinate. Medially with one pectinate, spine-like seta and 6–8 long, simple setae. Maxillary palp ca. 1.3× length of galea-lacinia; 2-segmented; palp segment II 1.2× length of segment I; setae on maxillary palp fine, simple, scattered over surface of segments I and II; apex of last segment without excavation at inner distolateral margin.

***Labium*** (Fig. 6h, i). Glossa basally broad, narrowing toward apex; much shorter than paraglossa; inner margin with two spine-like setae; apex with two long and one medium, robust setae; outer margin with three spine-like setae; ventral surface with fine, simple, scattered setae. Paraglossa broad, slightly curved inward; outer margin convex; apex rounded; with three long rows of long, robust, distally pectinate setae in apical area, five or six short, simple setae in anteromedial area and one short, simple seta in posteromedial area; dorsally with a row of four long, spine-like setae near inner margin. Labial palp with segment I 0.8× length of segments II and III combined. Segment I ventrally with short, fine, simple setae. Segment II with hook-like, distomedial protuberance; distomedial protuberance 0.9× width of base of segment III; ventral surface with short, simple setae; dorsally with one or two spine-like setae near outer margin. Segment III oblong; length 1.7× width; ventrally covered with short, spine-like, simple setae and short, fine, simple setae.

***Hind protoptera*** (Fig. 7i) absent.

***Foreleg*** (Fig. 7a–d). Ratio of foreleg segments 1.2:1.0:0.6:0.2. ***Femur*.** Length ca. 3× maximum width. Dorsal margin with a row of 18–25 curved, spine-like setae and a row of long, fine, simple setae; length of setae 0.21× maximum width of femur. Apex rounded, with a pair of spine-like setae and some short, stout setae. Stout, apically rounded setae scattered along ventral margin; femoral patch absent. ***Tibia*.** Dorsal margin with a row of short, spine-like setae. Ventral margin with a row of short, curved, spine-like setae, on apex some longer setae and a tuft of fine, simple setae. Anterior surface scattered with stout, lanceolate setae. Patellotibial suture present on basal 2/3 area. ***Tarsus*.** Dorsal margin with a row of short, spine-like setae. Ventral margin with a row of curved, spine-like setae and a row of short, stout setae near margin. Claw with one row of nine or ten denticles; distally pointed; with ca. four stripes; with long, subapical seta on posterior side and reduced, subapical seta on anterior side.

***Middle and hind legs*.** As foreleg, but middle leg with a reduced femoral patch and hind leg with a rather well developed femoral patch.

***Terga*** (Fig. 7e). Surface with irregular rows of U-shaped scale bases. Posterior margin of tergum IV with triangular, apically rounded spines, slightly longer than wide, and fine simple setae.

***Gills*** (Fig. 7f). Present on segments II–VII. Margin with small denticles intercalating fine simple setae. Tracheae extending from main trunk to inner and outer margins. Gill IV as long as length of segments V and 1/2 VI combined. Gill VII as long as length of segment VIII.

***Paraproct*** (Fig. 7g). Distally slightly expanded, with more than 40 stout, marginal spines. Surface scattered with U-shaped scale bases, fine, simple setae and micropores. Cercotractor with numerous small, marginal spines.

##### Etymology.

Dedicated to the indigenous Toraja people of Sulawesi, where the type locality is located.

##### Distribution.

Indonesia: Sulawesi (Fig. 21b).

##### Biological aspects.

The specimens were collected at an altitude of 1600 m in a tributary to Lake Lore.

### *Labiobaetisclaudiae* group of species (Kaltenbach and Gattolliat 2018)

The *claudiae* group is recognised by the following combination of characters: A) dorsal surface of labrum with submarginal arc of simple setae; B) labial palp segment II with rather narrow thumb-like distomedial protuberance; C) maxillary palp segment II without distolateral excavation, apex usually constricted; D) six pairs of gills (gill I absent); E) gills margin usually with both shorter and longer setae; F) hind protoptera absent; G) distolateral process at scape absent; H) femur dorsally with relatively short setae (length below 0.20× maximum width of femur); I) femur apically with stout setae on posterior side of foreleg and middle leg; J) femoral patch present on all legs.

The *L.claudiae* group is known from New Guinea only, it includes the following species:

*Labiobaetisacademicus* Kaltenbach, Surbakti & Kluge, 2021

*Labiobaetiscentralensis* Kaltenbach & Gattolliat, 2018 (new assignment, see discussion)

*Labiobaetisclaudiae* Kaltenbach & Gattolliat, 2018

*Labiobaetishattam* sp. nov.

*Labiobaetisstagnum* Kaltenbach & Gattolliat, 2018

*Labiobaetiswerneri* sp. nov.

### Key to the species of the *Labiobaetisclaudiae* group (larvae)

**Table:** 

1	Paraproct distally expanded (Fig. 9g)	**2**
–	Paraproct distally not expanded (Fig. 11f)	**4**
2	Anal margins of gills with both longer and shorter setae (Kaltenbach et al. 2021: fig. 4c, d)	***L.stagnum***
–	Anal margin of gills with short setae only (Fig. 9f).	**3**
3	Posterior margins of tergites with triangular spines, longer than wide (Fig. 9e); scape with stout setae (Fig. 9h).	***L.hattam* sp. nov.**
–	Posterior margins of tergites with triangular spines, wider than long (Kaltenbach and Gattolliat 2018: fig. 47e); scape without stout setae	***L.centralensis***
4	Posterior margins of tergites with triangular spines, wider than long (Fig. 11d)	***L.werneri* sp. nov.**
–	Posterior margins of tergites with triangular spines, longer than wide (Kaltenbach and Gattolliat 2018: fig. 9c)	**5**
5	Labial palp segment II with rather broad thumb-like distomedial protuberance (Kaltenbach et al. 2021: fig. 4f)	***L.claudiae***
–	Labial palp segment II with narrow thumb-like distomedial protuberance (Kaltenbach et al. 2021: fig. 1h)	***L.academicus***


#### Labiobaetis hattam sp. nov.

Taxon classificationAnimaliaEphemeropteraBaetidae

153A45BD-8742-5294-873B-82BB74382DFD

http://zoobank.org/A8E19D2A-874A-4BDC-BC26-EB900063EC91

##### Type material.

***Holotype*.** Indonesia • larva; Papua Barat, Fumato to Kebar, forest stream; 00°52'29"S, 132°46'06"E; 492 m; 06.xi.2013; leg. UNIPA team; BH030; on slide; GBIFCH00592775; MZB. ***Paratypes*.** Indonesia • 3 larvae; same data as holotype; 2 on slides; GenBank MW868311; GBIFCH00763707, GBIFCH00592704; 1 in alcohol; GBIFCH00515652; MZB, MZL.

##### Diagnosis.

**Figure 8.**
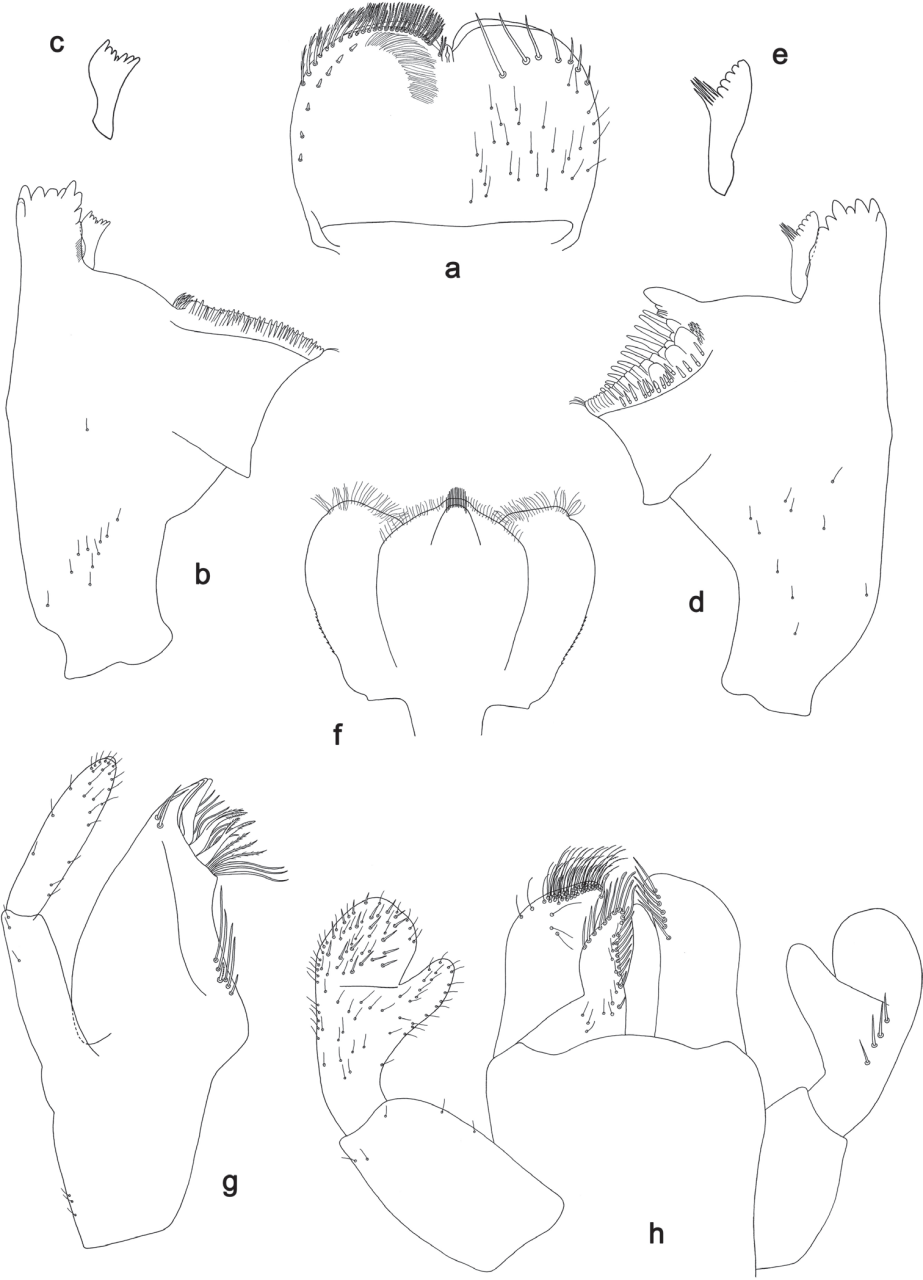
*Labiobaetishattam* sp. nov., larva morphology: **a** labrum **b** right mandible **c** right prostheca **d** left mandible **e** left prostheca **f** hypopharynx and superlinguae **g** maxilla **h** labium.

**Figure 9.**
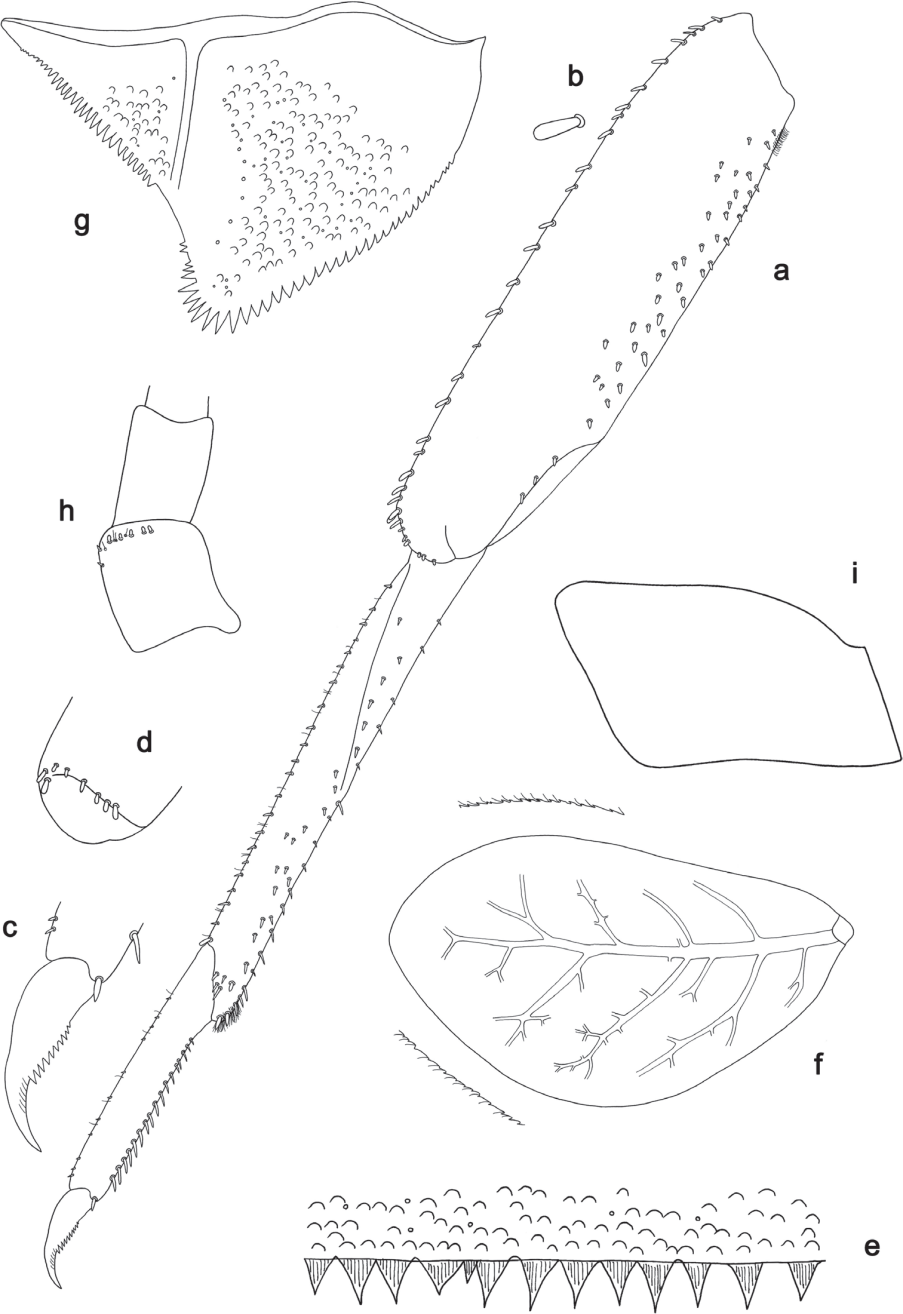
*Labiobaetishattam* sp. nov., larva morphology: **a** foreleg **b** seta on dorsal margin of femur **c** fore claw **d** apex of forefemur, posterior view **e** tergum IV **f** gill IV **g** paraproct **h** base of antenna **i** Metanotum.

**Larva.** Following combination of characters: A) dorsal surface of labrum with submarginal arc of one plus six long, simple setae (Fig. 8a); B) labial palp segment II with narrow, extended, distomedial protuberance, segment III slightly pentagonal (Fig. 8h); C) fore femur rather slender, length ca. 4× maximum width, dorsal margin with 19–23 spine-like setae (Fig. 9a); D) hind protoptera absent; E) six pairs of gills (gill I absent), margin with short setae only; F) paraproct distally slightly expanded, with 38–48 stout, marginal spines (Fig. 9g); G) Scape apically with stout setae (Fig. 9h).

##### Description.

**Larva** (Figs 8, 9, 18a, b). Body length 7.0–7.6 mm. Cerci and paracercus broken. Antenna: approx. 3× as long as head length.

***Colouration*** (Fig. 18a, b). Head, thorax and abdomen dorsally dark brown, with pattern as in Fig. 18a; fore protoptera brown with bright stripes. Thorax ventrally ecru, abdomen ventrally brown, with pattern as in Fig. 18b. Legs light brown; femur with dorsomedial and apical brown spots; tibia medially and tarsus proximally brown. Caudalii brown.

***Antenna*** (Fig. 9h) with scape and pedicel sub cylindrical, without distolateral process at scape. Scape apically with few stout setae.

***Labrum*** (Fig. 8a). Sub-rectangular, length 0.8× maximum width. Distal margin with medial emargination and a small process. Dorsally with medium, fine, simple setae scattered over surface; submarginal arc of setae composed of one plus six long, simple setae. Ventrally with marginal row of setae composed of anterolateral long, feathered setae and medial long, bifid, pectinate setae; ventral surface with ca. seven short, spine-like setae near lateral and anterolateral margin.

***Right mandible*** (Fig. 8b, c). Incisor and kinetodontium fused. Incisor with five denticles; kinetodontium with three denticles, inner margin of innermost denticle with a row of thin setae. Prostheca robust, apically denticulate. Margin between prostheca and mola almost straight. Tuft of setae at apex of mola present.

***Left mandible*** (Fig. 8d, e). Incisor and kinetodontium fused. Incisor with four denticles; kinetodontium with three denticles. Prostheca robust, apically with small denticles and comb-shaped structure. Margin between prostheca and mola almost straight. Subtriangular process long and slender, above level of area between prostheca and mola. Denticles of mola apically constricted. Tuft of setae at apex of mola present.

Both mandibles with lateral margins almost straight. Basal half with fine, simple setae scattered over dorsal surface.

***Hypopharynxandsuperlinguae*** (Fig. 8f). Lingua approx. as long as superlingua. Lingua longer than broad; distal half laterally slightly expanded; medial tuft of stout setae well developed and short. Superlinguae distally rounded; lateral margins rounded; fine, long, simple setae along distal margin.

***Maxilla*** (Fig. 8g). Galea-lacinia ventrally with two simple, apical setae under canines. Inner dorsal row of setae with three denti-setae, distal denti-seta tooth-like, middle and proximal denti-setae slender, bifid and pectinate. Medially with one pectinate, spine-like seta and four or five long, simple setae. Maxillary palp ca. 1.1× length of galea-lacinia; 2-segmented; palp segment II 1.2× length of segment I; setae on maxillary palp fine, simple, scattered over surface of segments I and II; apex of last segment without excavation at inner distolateral margin, apically constricted.

***Labium*** (Fig. 8h). Glossa basally broad, narrowing toward apex; shorter than paraglossa; inner margin with ten spine-like setae, increasing in length distally; apex with three long, robust, pectinate setae and one short, robust seta; outer margin with six or seven spine-like setae; ventral surface with fine, simple, scattered setae. Paraglossa sub-rectangular, curved inward; apex rounded; with three rows of long, robust, distally pectinate setae in apical area and two or three short, simple setae in anteromedial area; dorsally with a row of six or seven long, spine-like setae near inner margin. Labial palp with segment I 0.9× length of segments II and III combined. Segment I ventrally with short, fine, simple setae. Segment II with narrow, extended, distomedial protuberance; distomedial protuberance 0.6× width of base of segment III; ventral surface with short, simple setae; dorsally with three or four spine-like setae near outer margin. Segment III slightly pentagonal; length 0.9× width; ventrally covered with short, spine-like, simple setae and short, fine, simple setae.

***Hind protoptera*** (Fig. 9i) absent.

***Foreleg*** (Fig. 9a–d). Ratio of foreleg segments 1.3:1.0:0.6:0.2. ***Femur*.** Length ca. 4× maximum width. Dorsal margin with a row of 19–23 curved, spine-like, apically rounded setae; length of setae 0.1× maximum width of femur. Apex rounded, with a pair of spine-like setae and some short, stout setae. Many stout, lanceolate setae scattered along ventral margin; femoral patch present. On posterior side apically with stout setae. ***Tibia*.** Dorsal margin with a row of short, spine-like setae and fine, simple setae. Ventral margin with a row of short, curved, spine-like setae, on apex some longer setae and a tuft of fine, simple setae. Anterior surface scattered with stout, lanceolate setae. Patellotibial suture present on basal 1/2. ***Tarsus*.** Dorsal margin with a row of short, spine-like setae and fine, simple setae. Ventral margin with a row of curved, spine-like setae. Claw with one row of 10–12 denticles; distally pointed; with ca. six stripes; subapical setae absent.

***Middle and hind legs*** (Fig. 9d). As foreleg, also with femoral patch. Stout setae on apex of posterior side present on middle leg and absent on hind leg.

***Terga*** (Fig. 9e). Surface with irregular rows of U-shaped scale bases and scattered micropores. Posterior margin of tergum IV with triangular spines, longer than wide.

***Gills*** (Fig. 9f). Present on segments II–VII. Margin with small denticles intercalating fine simple setae. Tracheae extending from main trunk to inner and outer margins. Gill IV as long as length of segments V and VI combined. Gill VII as long as length of segments VIII and 2/3 IX combined.

***Paraproct*** (Fig. 9g). Distally slightly expanded, with 38–48 stout, marginal spines. Surface scattered with U-shaped scale bases and micropores. Cercotractor with numerous small, marginal spines.

##### Etymology.

Dedicated to the indigenous Hattam people from West Papua.

##### Distribution.

Indonesia: Papua Barat (Fig. 21c).

##### Biological aspects.

The specimens were collected in a forest stream at an altitude of 500 m.

#### Labiobaetis werneri sp. nov.

Taxon classificationAnimaliaEphemeropteraBaetidae

BAC6CCF1-584F-5A06-B3B5-B5E770D3E334

http://zoobank.org/24BA8E1B-868C-408D-9EAC-2952CA612F18

##### Type material.

***Holotype*.** Papua New Guinea • larva; Gulf, Marawaka, Mala; 07°05'40"S, 145°44'28"E; 1400 m; 11.xi.2006; leg. Balke and Kinibel; (PNG 90); on slide; GenBank MW868307; GBIFCH00763699; ZSM. ***Paratypes*.** Papua New Guinea • 1 larva; same data as holotype; in alcohol; GBIFCH00515645; MZL • 1 larva; Eastern Highlands, Marawaka, Ande; 07°01'42"S, 145°49'48"E; 1700–1800 m; 09.xi.2006; leg. Balke and Kinibel; (PNG 87); on slide; GenBank MW868309; GBIFCH00763603; MZL • 10 larvae; Morobe, Wagau, Herzog Mts; 06°51'04"S, 146°48'04"E; 1150 m; 19.xi.2006; leg. Balke and Kinibel; (PNG 102); 1 on slide; GBIFCH00592773; MZL; 9 in alcohol; GenBank MW868308; GBIFCH00763700, GBIFCH00515644, GBIFCH00515651; MZL • 1 larva; Morobe, Garaina; 07°51'02"S, 147°07'00"E; 720 m; vi.2008; leg. Ibalim and Sosanika; (PNG216); in alcohol; GBIFCH00829895; MZL.

##### Diagnosis.

**Figure 10.**
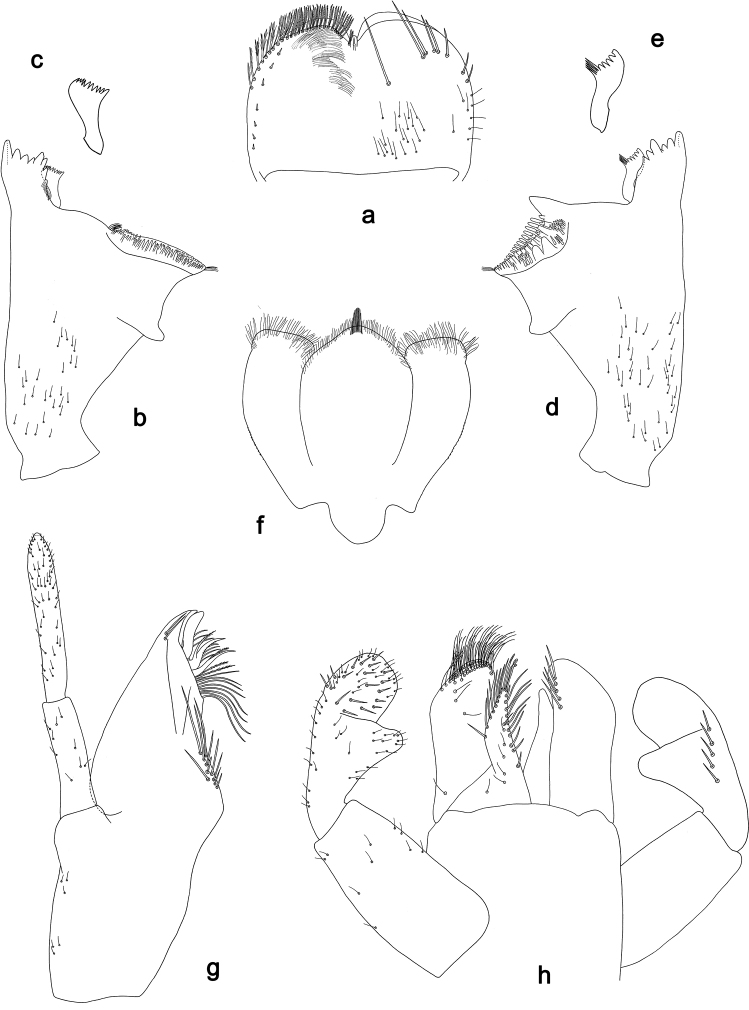
*Labiobaetiswerneri* sp. nov., larva morphology: **a** labrum **b** right mandible **c** right prostheca **d** left mandible **e** left prostheca **f** hypopharynx and superlingua **g** maxilla **h** labium.

**Figure 11.**
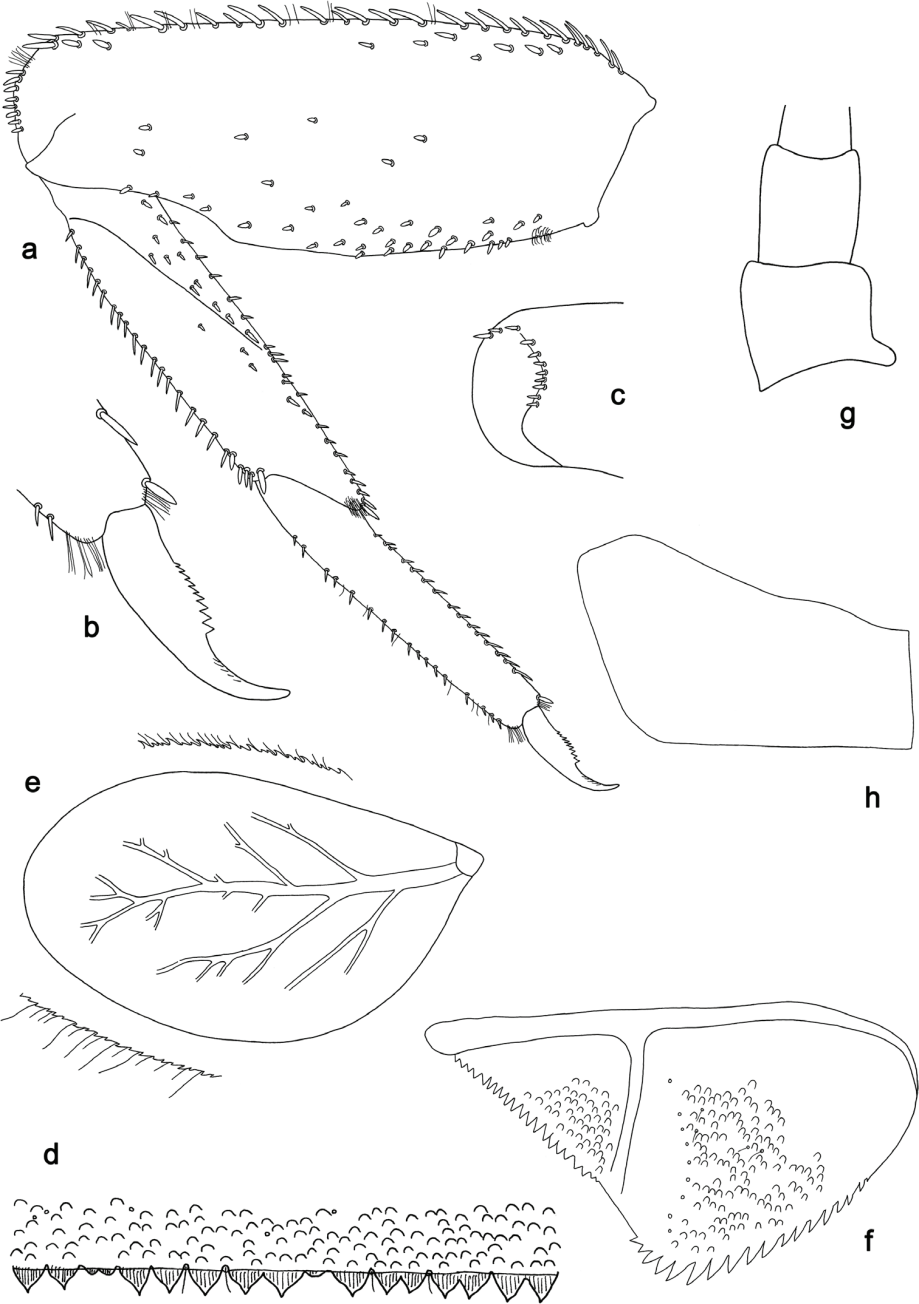
*Labiobaetiswerneri* sp. nov., larva morphology: **a** foreleg **b** fore claw **c** apex of forefemur, posterior view **d** tergum IV **e** gill IV **f** paraproct **g** base of antenna **h** metanotum.

**Larva.** Following combination of characters: A) dorsal surface of labrum with submarginal arc of one plus six or seven long, simple setae (Fig. 10a); B) labial palp segment II with rather narrow thumb-like, distomedial protuberance, segment III sub-rectangular (Fig. 10h); C) fore femur rather broad, length 2.7× maximum width, dorsal margin with 25–33 spine-like setae plus additional setae near margin (Fig. 11a); D) hind protoptera absent; E) six pairs of gills (gill I absent), anal margin with both short and long setae (Fig. 11e); F) paraproct distally not expanded, with 18–28 stout, marginal spines (Fig. 11f).

##### Description.

**Larva** (Figs 10, 11, 19a, b). Body length 5.7–6.2 mm. Cerci broken, paracercus ca. 0.4× body length. Antenna: approx. 2.5× as long as head length.

***Colouration*** (Fig. 19a, b). Head, thorax and abdomen dorsally brown, with pattern as in Fig. 19a. Head, thorax and abdomen ventrally light brown, abdominal sternites VI–IX darker, as in Fig. 19b. Legs light brown; femur with dorsomedial and apical brown spots; tarsus distally brown. Caudalii light brown.

***Antenna*** (Fig. 11g) with scape and pedicel sub cylindrical, without distolateral process at scape.

***Labrum*** (Fig. 10a). Sub-rectangular, length 0.7× maximum width. Distal margin with medial emargination and a small process. Dorsally with medium, fine, simple setae scattered over surface; submarginal arc of setae composed of one plus six or seven long, simple setae. Ventrally with marginal row of setae composed of anterolateral long, feathered setae and medial long, bifid, pectinate setae; ventral surface with ca. seven short, spine-like setae near lateral and anterolateral margin.

***Right mandible*** (Fig. 10b, c). Incisor and kinetodontium fused. Incisor with four denticles; kinetodontium with three denticles, inner margin of innermost denticle with a row of thin setae. Prostheca robust, apically denticulate. Margin between prostheca and mola almost straight. Tuft of setae at apex of mola present.

***Left mandible*** (Fig. 10d, e). Incisor and kinetodontium fused. Incisor with four denticles; kinetodontium with three denticles. Prostheca robust, apically with small denticles and comb-shaped structure. Margin between prostheca and mola straight. Subtriangular process long and slender, above level of area between prostheca and mola. Denticles of mola apically constricted. Tuft of setae at apex of mola present.

Both mandibles with lateral margins almost straight. Basal half with fine, simple setae scattered over dorsal surface.

***Hypopharynxandsuperlinguae*** (Fig. 10f). Lingua approx. as long as superlinguae. Lingua longer than broad; distal half laterally slightly expanded; medial tuft of stout setae well developed and short. Superlinguae distally rounded; lateral margins rounded; fine, long, simple setae along distal margin.

***Maxilla*** (Fig. 10g). Galea-lacinia ventrally with two simple, apical setae under canines. Inner dorsal row of setae with three denti-setae, distal denti-seta tooth-like, middle and proximal denti-setae slender, bifid and pectinate. Medially with one pectinate, spine-like seta and 7–9 long, simple setae. Maxillary palp ca. 1.3× length of galea-lacinia; 2-segmented; palp segment II 1.5× length of segment I; setae on maxillary palp fine, simple, scattered over surface of segments I and II; apex of last segment without excavation at inner distolateral margin, apically constricted.

***Labium*** (Fig. 10h). Glossa basally broad, narrowing toward apex; shorter than paraglossa; inner margin with nine spine-like setae, increasing in length distally; apex with three long, robust, pectinate setae and one short, robust seta; outer margin with seven or eight spine-like setae; ventral surface with fine, simple, scattered setae. Paraglossa sub-rectangular, curved inward; apex rounded; with three rows of long, robust, distally pectinate setae in apical area, three or four short, simple setae in anteromedial area and one short, simple seta in posterolateral area; dorsally with a row of five long, spine-like setae near inner margin. Labial palp with segment I 1.1× length of segments II and III combined. Segment I ventrally with short, fine, simple setae. Segment II with narrow thumb-like, distomedial protuberance; distomedial protuberance 0.5× width of base of segment III; ventral surface with short, simple setae; dorsally with 3–5 spine-like setae near outer margin. Segment III sub-rectangular; length 1.1× width; ventrally covered with short, spine-like, simple setae and short, fine, simple setae.

***Hind protoptera*** (Fig. 11h) absent.

***Foreleg*** (Fig. 11a–c). Ratio of foreleg segments 1.6:1.0:0.9:0.3. ***Femur*.** Length 2.7× maximum width. Dorsal margin with a row of 25–33 curved, spine-like setae and additional setae near margin; length of setae 0.16× maximum width of femur. Apex rounded, with a pair of spine-like setae and some short, stout setae. Many stout, lanceolate setae scattered along ventral margin and some scattered on surface; femoral patch present. On posterior side apically with stout setae. ***Tibia*.** Dorsal margin with a row of short to medium, spine-like setae. Ventral margin with a row of short, curved, spine-like setae, on apex some longer setae and a tuft of fine, simple setae. Anterior surface scattered with stout, lanceolate setae. Patellotibial suture present on basal 1/2. ***Tarsus*.** Dorsal margin with a row of short, spine-like setae and fine, simple setae. Ventral margin with a row of curved, spine-like setae. Claw with one row of 9–12 denticles; distally pointed; with ca. five stripes; subapical setae absent.

***Middle and hind legs*** (Fig. 11c). As foreleg, also with femoral patch. Stout setae on apex of posterior side present on middle leg and absent on hind leg.

***Terga*** (Fig. 11d). Surface with irregular rows of U-shaped scale bases and scattered micropores. Posterior margin of tergum IV with triangular spines, wider than long.

***Gills*** (Fig. 11e). Present on segments II–VII. Costal margin with small denticles intercalating short, fine simple setae; anal margin with small denticles, intercalating both short and long, fine, simple setae. Tracheae extending from main trunk to inner and outer margins. Gill IV as long as length of segments V and 2/3 VI combined. Gill VII as long as length of segments VIII and 1/2 IX combined.

***Paraproct*** (Fig. 11f). Distally not expanded, with 18–28 stout, marginal spines. Surface scattered with U-shaped scale bases and fine, simple setae. Cercotractor with numerous small, marginal spines.

##### Etymology.

Dedicated to Werner Horzel, the late stepfather of the first author.

##### Distribution.

Papua New Guinea (Fig. 21c).

##### Biological aspects.

The specimens were collected at altitudes from 1150 m to 1800 m.

### *Labiobaetisdendrisetis* group of species (Kaltenbach et al. 2020)

The *dendrisetis* group can be recognised by the following combination of characters: A) dorsal surface of labrum with submarginl arc of dendritic setae; B) labial palp segment II with narrow thumb-like protuberance; C) labial palp segment III broad, rounded; D) seven pairs of gills.

The *L.dendrisetis* group is present in New Guinea and the Philippines; it includes the following species:

*Labiobaetisdalisay* Kaltenbach, Garces & Gattolliat, 2020

*Labiobaetisdendrisetis* Kaltenbach & Gattolliat, 2018

#### Labiobaetis dendrisetis

Taxon classificationAnimaliaEphemeropteraBaetidae

D54C3BE2-607F-5ED8-94AC-3DD13F9F9A88

 Kaltenbach and Gattolliat 2018: figs 48, 49.

##### Material examined.

Papua New Guinea • 9 larvae; Central Prov., Kokoda Trek; 09°00'20"S, 147°44'15"E; 1390 m; i.2008; leg. Posman; (PNG 173); 2 on slides; GenBank MW868310; GBIFCH00763706, 592769; MZL; 7 in alcohol; GBIFCH00515630, GBIFCH00515650; MZL • 1 larva; Central Prov., Woitape; 08°31'35"S, 147°14'06"E; 1600 m; i.2008; leg. Posman; (PNG 165); on slide; GBIFCH00515631; MZL • 1 larva; Central Prov., Woitape; 08°31'17"S, 147°13'41"E; 1700 m; i.2008; leg. Posman; (PNG 166); on slide; GBIFCH00592705; MZL.

##### Diagnosis.

**Larva.** Following combination of characters: A) dorsal surface of labrum with submarginal arc of long, dendritic setae setae (Kaltenbach and Gattolliat 2018: fig. 48a, b); B) labial palp segment II with short, narrow, thumb-like, distomedial protuberance, segment III broad, rounded (Kaltenbach and Gattolliat 2018: fig. 48k); C) mandibles with outermost incisor blade-like (Kaltenbach and Gattolliat 2018: fig. 48g); D) fore femur length ca. 3× maximum width, dorsal margin with ca. 20 curved, spine-like setae and proximally a partial second row of spine-like setae near margin (Fig. 14a); D) hind protoptera present; E) seven pairs of gills; F) scape without distolateral process (Fig. 17c).

Due to the limited material in the type series (holotype and one paratype), a few parts of the original description were missing, incomplete or have to be corrected:

##### Complementary description.

**Figure 12.**
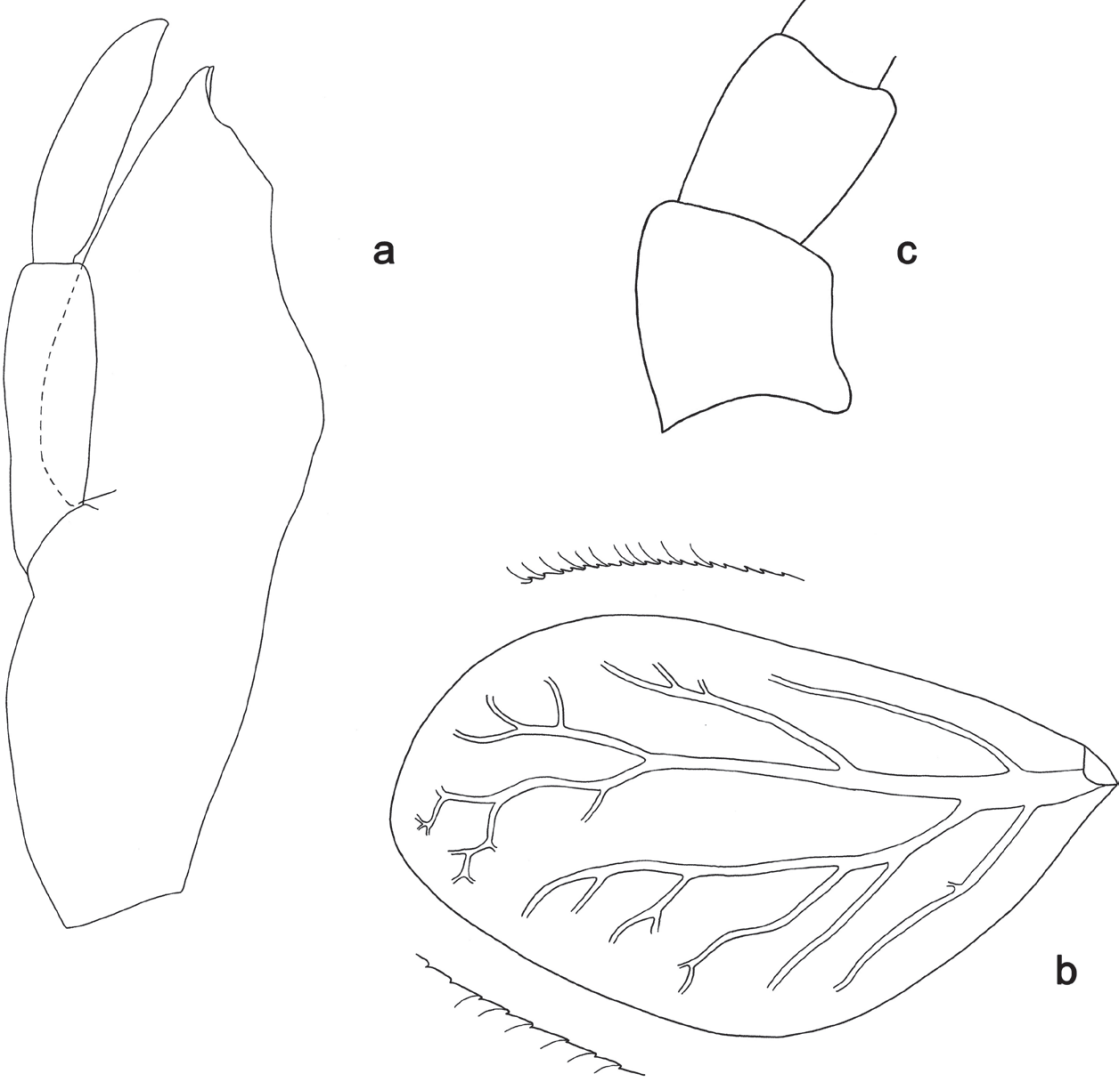
*Labiobaetisdendrisetis*, larva morphology: **a** maxilla **b** base of antenna **c** gill IV.

**Larva** (Figs 12, 19c, d). Body length 4.1–5.3 mm. Cerci broken, paracercus ca. half body length.

***Colouration*** (Fig. 19c, d). Head dorsally ochreous, thorax and abdomen dorsally brown, with pattern as in Fig. 19c. Head, thorax and abdomen ventrally ecru, abdominal sternites VI–VIII dark brown (Fig. 19d). Legs light brown, femur with distomedial brown streak. Caudalii light brown.

***Maxillary palp*** (Fig. 12a) ca. 1.1× length of galea-lacinia; 2-segmented; palp segment II approx. as long as segment I; apex of last segment with slight excavation at inner distolateral margin, apically rounded.

***Gills*** (Fig. 12b). Present on segments I–VII. Margin with small denticles intercalating fine simple setae. Tracheae extending from main trunk to inner and outer margins. Gill I as long as length of segment II. Gill IV as long as length of segments V, VI and 2/3 VII combined. Gill VII as long as length of segments VIII, IX and 1/3 X combined.

##### Distribution.

Papua New Guinea (Fig. 21c).

### *Labiobaetisseramensis* group of species (Kaltenbach and Gattolliat 2019)

The *seramensis* group is recognised by the following combination of characters: A) dorsal surface of labrum with submarginal arc of simple setae; B) labial palp segment II with narrow or rather narrow, thumb-like distomedial protuberance, segment III broad, rounded; C) femur dorsally with dense setation; middle and hind leg with reduced femoral patch; D) six pairs of gills (gill I absent); E) hind protoptera absent; F) distolateral process at scape absent.

The *L.seramensis* group is reported from Seram (Indonesia) and New Guinea (Indonesia: Papua Barat), it includes the following species:

*Labiobaetisarfak* sp. nov. (New Guinea)

*Labiobaetisonim* sp. nov. (New Guinea)

*Labiobaetisseramensis* Kaltenbach & Gattolliat, 2019 (Seram)

*Labiobaetiswahai* Kaltenbach & Gattolliat, 2019 (Seram)

### Key to the species of the *Labiobaetisseramensis* group (larvae)

**Table:** 

1	Dorsal margin of femur with row of more than 70 long, curved, spine-like setae (Fig. 16a); posterior margin of tergite IV with discontinued row of triangular spines (Fig. 16e)	***L.onim* sp. nov.**
–	Dorsal margin of femur with less than 25 curved, spine-like setae; posterior margin of tergite IV with continued row of triangular spines (Fig. 14e)	**2**
2	Abdominal tergites dark brown, segments V and VI yellow brown (Fig. 20a); posterolateral margins of tergites VIII and IX with two long, pointed spines (Fig. 14f)	***L.arfak* sp. nov.**
–	Abdominal tergites light brown (Kaltenbach and Gattolliat 2019: figs. 50c, d); posterolateral margins of tergites VIII and IX without long spines.	**3**
3	Posterior margins of tergites with triangular spines, wider than long (Kaltenbach and Gattolliat 2019: fig. 39c); paraproct distally not expanded (Kaltenbach and Gattolliat 2019: fig. 39d)	***L.seramensis***
–	Posterior margins of tergites with triangular spines, longer than wide (Kaltenbach and Gattolliat 2019: fig. 41c); paraproct distally expanded (Kaltenbach and Gattolliat 2019: fig. 41d)	***L.wahai***


#### Labiobaetis arfak sp. nov.

Taxon classificationAnimaliaEphemeropteraBaetidae

80851647-0FBD-5D3D-80B2-E1872AFD2B37

http://zoobank.org/EBC7E07E-1A03-4BD4-B98F-85F0E68CB4B6

##### Type material.

***Holotype*.** Indonesia • larva; Papua Barat, River Je, Loc. Arfak, East of Amber village; 01°10'59"S, 133°56'51"E; 1200 m; 16.vi.2016, leg. Sumoked; on slide; GBIFCH00592770; MZB. ***Paratypes*.** Indonesia • 27 larvae; same data as holotype; 4 on slides; GenBank MW868312, MW868313; GBIFCH00763714, GBIFCH00763715, GBIFCH00592767, GBIFCH00829897; MZL; 23 in alcohol; GBIFCH00515626, GBIFCH00515648; MZB, MZL.

##### Diagnosis.

**Figure 13.**
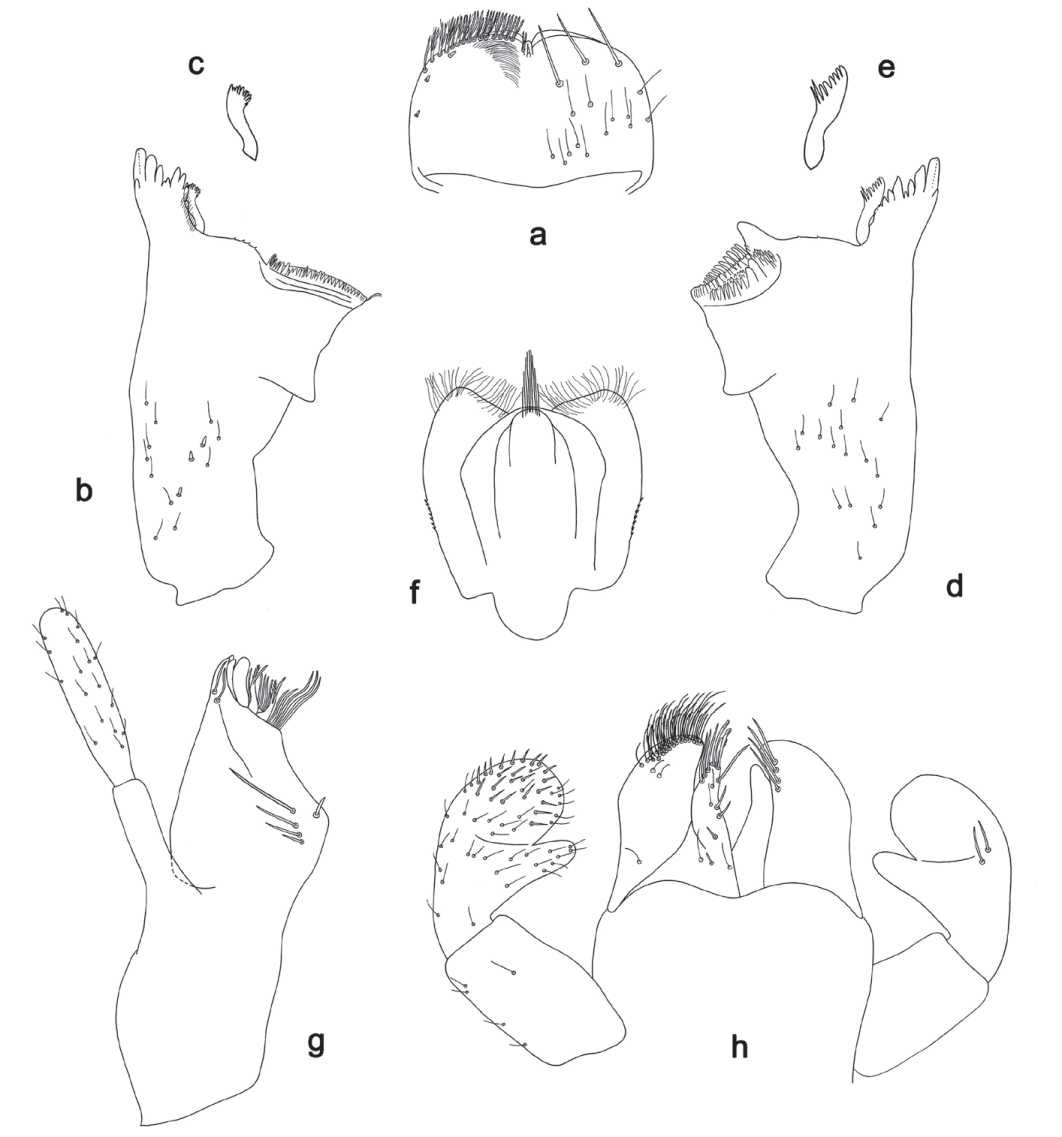
*Labiobaetisarfak* sp. nov., larva morphology: **a** labrum **b** right mandible **c** right prostheca **d** left mandible **e** left prostheca **f** hypopharynx and superlingua **g** maxilla **h** labium.

**Figure 14.**
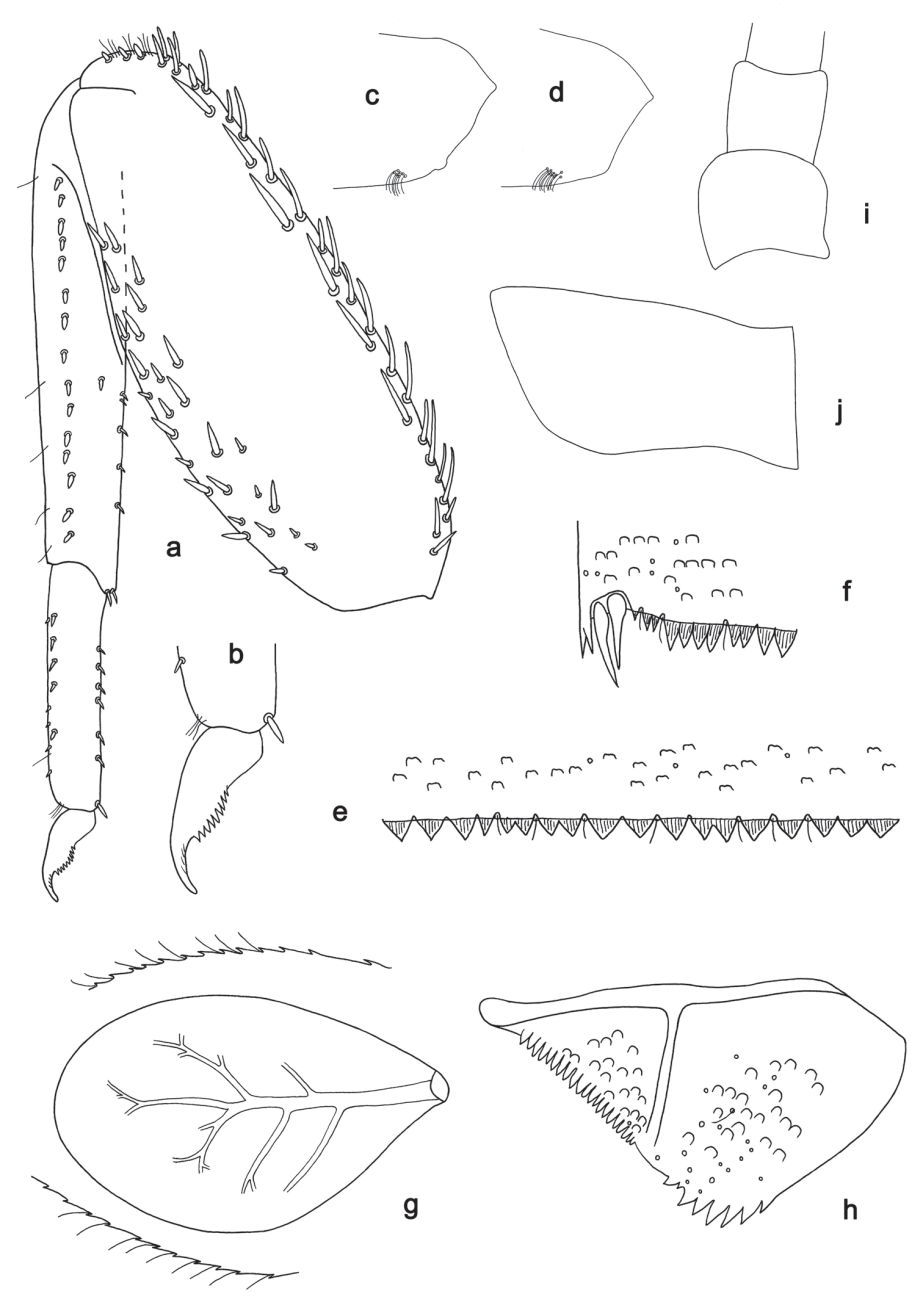
*Labiobaetisarfak* sp. nov., larva morphology: **a** foreleg **b** fore claw **c** base of middle femur **d** base of hind femur **e** tergum IV **f** tergum IX **g** gill IV **h** paraproct **i** base of antenna **j** metanotum.

**Larva.** Following combination of characters: A) dorsal surface of labrum with submarginal arc of one plus two long, simple setae (Fig. 13a); B) labial palp segment II with narrow thumb-like, distomedial protuberance, segment III broad, rounded (Fig. 13h); C) fore femur rather broad, length 2.7× maximum width, dorsal margin with 17–20 spine-like setae plus a second row of spine-like setae near margin (Fig. 14a); D) hind protoptera absent; E) six pairs of gills (gill I absent); F) tergites VIII and IX posterolaterally with two long spines (Fig. 14f); G) paraproct distally not expanded, with ca. eight stout, marginal spines (Fig. 14h).

##### Description.

**Larva** (Figs 13, 14, 20a, b). Body length 3.3–4.4 mm. Cerci ca. 2/3 of body length, paracercus ca. 2/3 of cerci length. Antenna: approx. 2.5× as long as head length.

***Colouration*** (Fig. 20a, b). Head, thorax and abdomen dorsally dark brown, abdominal tergites V and VI yellow brown (Fig. 20a). Thorax ventrally ecru, abdomen ventrally brown, with pattern as in Fig. 20b. Legs light brown; femur dorsally and ventrally dark brown, basally and distomedially with dark brown areas (Fig. 20b). Caudalii light brown, basally brown.

***Antenna*** (Fig. 14i) with scape and pedicel sub cylindrical, without distolateral process at scape.

***Labrum*** (Fig. 13a). Sub-rectangular, length 0.6× maximum width. Distal margin with medial emargination and a small process. Dorsally with medium, fine, simple setae scattered over surface; submarginal arc of setae composed of one plus two long, simple setae. Ventrally with marginal row of setae composed of anterolateral long, feathered setae and medial long, bifid, pectinate setae; ventral surface with ca. three short, spine-like setae near lateral and anterolateral margin.

***Right mandible*** (Fig. 13b, c). Incisor and kinetodontium fused. Incisor with five denticles; kinetodontium with three denticles, inner margin of innermost denticle with a row of thin setae. Prostheca robust, apically denticulate. Margin between prostheca and mola convex, with minute denticles. Tuft of setae at apex of mola present.

***Left mandible*** (Fig. 13d, e). Incisor and kinetodontium fused. Incisor with five denticles; kinetodontium with three denticles. Prostheca robust, apically with small denticles and comb-shaped structure. Margin between prostheca and mola almost straight, with few minute denticles. Subtriangular process long and slender, above level of area between prostheca and mola. Denticles of mola apically constricted. Tuft of setae at apex of mola absent.

Both mandibles with lateral margins almost straight. Basal half with fine, simple setae scattered over dorsal surface.

***Hypopharynxandsuperlinguae*** (Fig. 13f). Lingua shorter than superlingua. Lingua longer than broad; distal half laterally slightly expanded; medial tuft of stout setae well developed and long. Superlinguae distally rounded; lateral margins rounded; fine, long, simple setae along distal margin.

***Maxilla*** (Fig. 13g). Galea-lacinia ventrally with two simple, apical setae under canines. Inner dorsal row of setae with three denti-setae, distal denti-seta tooth-like, middle and proximal denti-setae slender, bifid and pectinate. Medially with one spine-like seta and four long, simple setae. Maxillary palp ca. 1.3× length of galea-lacinia; 2-segmented; palp segment II 1.5× length of segment I; setae on maxillary palp fine, simple, scattered over surface of segment II; apex of last segment without excavation at inner distolateral margin, apically rounded.

***Labium*** (Fig. 13h). Glossa basally broad, narrowing toward apex; shorter than paraglossa; inner margin with 3–5 spine-like setae, distalmost seta much longer; apex with two long and one medium robust, pectinate setae; outer margin with three or four spine-like setae; ventral surface with fine, simple, scattered setae. Paraglossa sub-rectangular, curved inward; apex rounded; with three rows of long, robust, distally pectinate setae in apical area, one or two short, simple setae in anteromedial area and one short, simple seta in posterolateral area; dorsally with a row of four long, spine-like setae near inner margin. Labial palp with segment I 0.9× length of segments II and III combined. Segment I ventrally with short, fine, simple setae. Segment II with narrow thumb-like, distomedial protuberance; distomedial protuberance 0.4× width of base of segment III; ventral surface with short, simple setae; dorsally with two or three spine-like setae near outer margin. Segment III broad, rounded; length 0.9× width; ventrally covered with short, spine-like, simple setae and short, fine, simple setae.

***Hind protoptera*** (Fig. 14j) absent.

***Foreleg*** (Fig. 14a, b). Ratio of foreleg segments 1.3:1.0:0.5:0.2. ***Femur*.** Length 2.7× maximum width. Dorsal margin with a row of 17–20 curved, spine-like setae and a second row of spine-like setae near margin; length of setae 0.25× maximum width of femur. Apex rounded, with one or two pairs of spine-like setae and some short, stout setae. Many stout, lanceolate setae scattered along ventral margin; femoral patch absent. ***Tibia*.** Dorsal margin with a row of short, spine-like setae and fine, simple setae. Ventral margin with a row of short, curved, spine-like setae. Anterior surface scattered with few stout, lanceolate setae. Patellotibial suture present on basal 1/2 area. ***Tarsus*.** Dorsal margin with a row of short, spine-like setae. Ventral margin with a row of curved, spine-like setae. Claw with one row of 9–11 denticles; distally pointed; with ca. five stripes; subapical setae absent.

***Middle and hind legs*** (Fig. 14c, d). As foreleg, but with reduced femoral patch.

***Terga*** (Fig. 14e, f). Surface with irregular rows of U-shaped scale bases and scattered micropores. Posterior margin of tergum IV with triangular spines, wider than long. Posterolateral margins of terga VIII and IX with two long, pointed spines.

***Gills*** (Fig. 14g). Present on segments II–VII. Margin with small denticles intercalating fine simple setae. Tracheae extending from main trunk to inner and outer margins. Gill IV as long as length of segments V and 2/3 VI combined. Gill VII as long as length of segments VIII and 2/3 IX combined.

***Paraproct*** (Fig. 14h). Distally not expanded, with ca. eight stout, marginal spines. Surface scattered with U-shaped scale bases, micropores and fine, simple setae. Cercotractor with numerous small, marginal spines.

##### Etymology.

Dedicated to the indigenous Arfak people of Papua Barat, where the type locality is located.

##### Distribution.

Indonesia: Papua Barat (Fig. 21c).

##### Biological aspects.

The specimens were collected at an altitude of 1200 m, together with *L.onim* sp. nov.

#### Labiobaetis onim sp. nov.

Taxon classificationAnimaliaEphemeropteraBaetidae

2C8338CC-C435-595E-8B54-1E66BAF3A317

http://zoobank.org/ADFC666F-D838-4FC3-AA6B-1D473939F07E

##### Type material.

***Holotype*.** Indonesia • larva; Papua Barat, River Je, Loc. Arfak, East of Amber village; 01°10'59"S, 133°56'51"E; 1200 m; 16.vi.2016, leg. Sumoked; on slide; GBIFCH00763713; MZB. ***Paratypes*.** Indonesia • 2 larvae; same data as holotype; 2 on slides; GBIFCH00515649, GBIFCH00592706; MZB, MZL.

##### Diagnosis.

**Figure 15.**
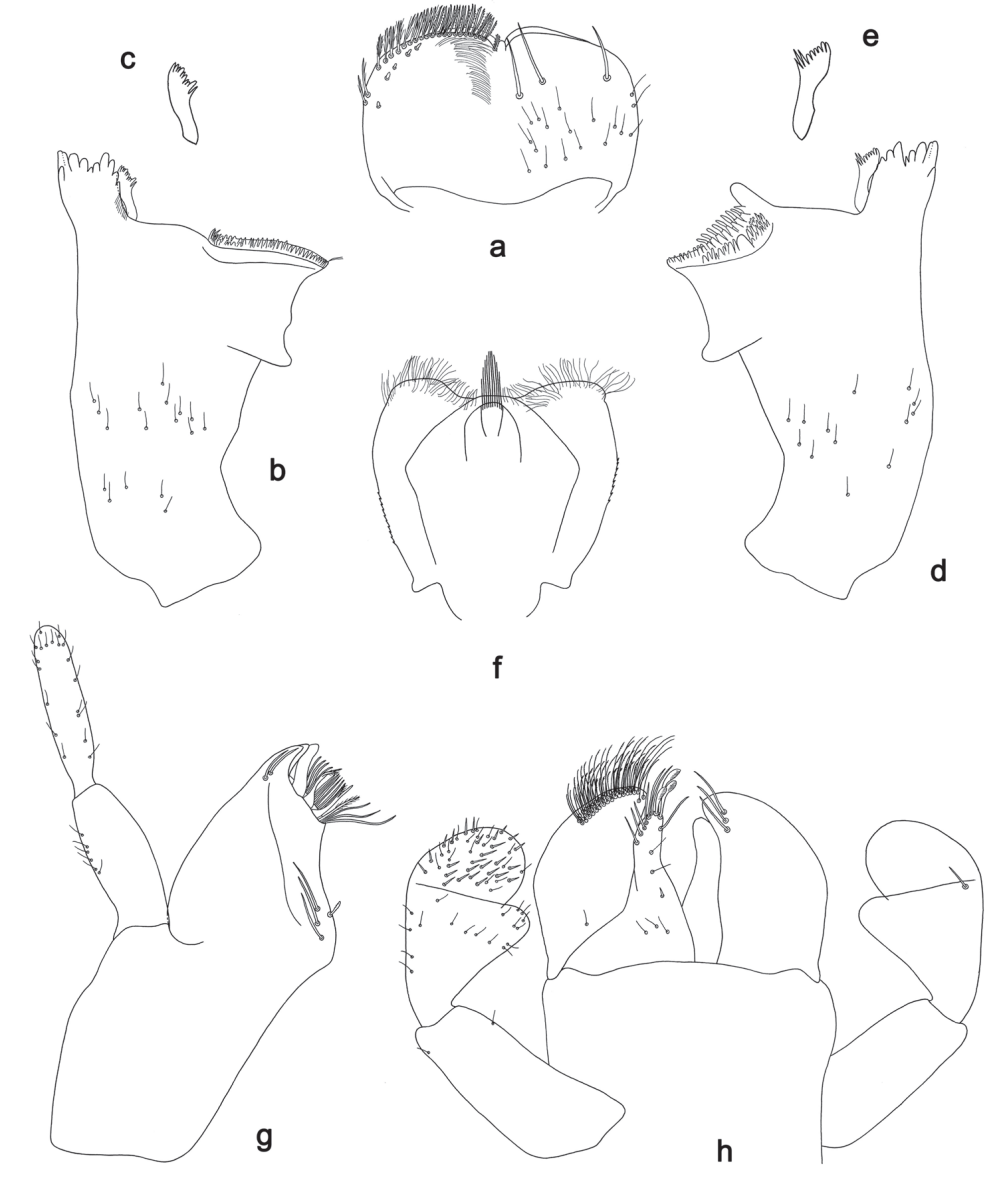
*Labiobaetisonim* sp. nov., larva morphology: **a** labrum **b** right mandible **c** right prostheca **d** left mandible **e** left prostheca **f** hypopharynx and superlingua **g** maxilla **h** labium.

**Figure 16:**
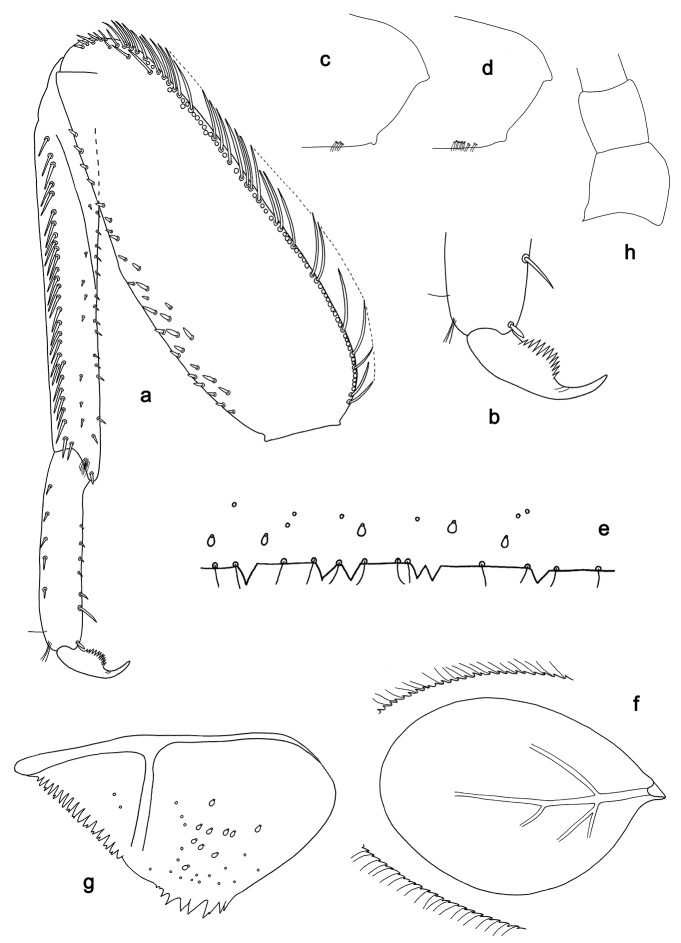
. *Labiobaetisonim* sp. nov., larva morphology: **a** Foreleg **b** Fore claw **c** Base of middle femur **d** Base of hind femur **e** Tergum IV **f** Gill IV **g** Paraproct **h** Base of antenna.

**Larva.** Following combination of characters: A) dorsal surface of labrum with submarginal arc of one plus two long, simple setae (Fig. 15a); B) labial palp segment II with short thumb-like (atypical for the group), distomedial protuberance, segment III broad, rounded (Fig. 15h); C) fore femur rather broad, length ca. 3× maximum width, dorsal margin with more than 70 long, curved spine-like setae plus some additional spine-like setae near margin (Fig. 16a); D) hind protoptera absent; E) six pairs of gills (gill I absent); F) paraproct distally not expanded, with 8–12 stout, marginal spines (Fig. 16g).

##### Description.

**Larva** (Figs 15, 16, 20c, d). Body length 5.6–5.9 mm. Cerci ca. 1/2 of body length, paracercus ca. 2/3 of cerci length. Antenna: approx. twice as long as head length.

***Colouration*** (Fig. 20c, d). Head dorsally light brown, thorax and abdomen dorsally dark brown, with light brown pattern on thorax as in Fig. 20c, abdominal segment I light brown and abdominal segments V–VII orange. Head, thorax, and abdomen ventrally light brown, with pattern as in fig. 20d, abdominal segments V–VII light orange and abdominal segments VIII–X dark brown. Legs ecru, caudalii ecru.

***Antenna*** (Fig. 16h) with scape and pedicel sub cylindrical, without distolateral process at scape.

***Labrum*** (Fig. 15a). Sub-rectangular, length 0.6× maximum width. Distal margin with medial emargination and a small process. Dorsally with medium, fine, simple setae scattered over surface; submarginal arc of setae composed of one plus two long, simple setae; large distance between both arc setae. Ventrally with marginal row of setae composed of anterolateral long, feathered setae and medial long, bifid, pectinate setae; ventral surface with ca. five short, spine-like setae near lateral and anterolateral margin.

***Right mandible*** (Fig. 15b, c). Incisor and kinetodontium fused. Incisor with six denticles; kinetodontium with three denticles, inner margin of innermost denticle with a row of thin setae. Prostheca robust, apically denticulate. Margin between prostheca and mola slightly convex. Tuft of setae at apex of mola present.

***Left mandible*** (Fig. 15d, e). Incisor and kinetodontium fused. Incisor with five denticles; kinetodontium with three denticles. Prostheca robust, apically with small denticles and comb-shaped structure. Margin between prostheca and mola straight. Subtriangular process long and slender, above level of area between prostheca and mola. Denticles of mola apically constricted. Tuft of setae at apex of mola absent.

Both mandibles with lateral margins almost straight. Basal half with fine, simple setae scattered over dorsal surface.

***Hypopharynxandsuperlinguae*** (Fig. 15f). Lingua shorter than superlingua. Lingua longer than broad; distal half laterally slightly expanded; medial tuft of stout setae well developed and long. Superlinguae distally rounded; lateral margins rounded; fine, long, simple setae along distal margin.

***Maxilla*** (Fig. 15g). Galea-lacinia ventrally with two simple, apical setae under canines. Inner dorsal row of setae with three denti-setae, distal denti-seta tooth-like, middle and proximal denti-setae slender, bifid and pectinate. Medially with one pectinate, spine-like seta and three long, simple setae. Maxillary palp ca. 1.3× length of galea-lacinia; 2-segmented; palp segment II 1.1× length of segment I; setae on maxillary palp fine, simple, scattered over surface of segments I and II; apex of last segment without excavation at inner distolateral margin, apically rounded.

***Labium*** (Fig. 15h). Glossa basally broad, narrowing toward apex; shorter than paraglossa; inner margin with one long, spine-like seta; apex with two long and one medium robust, pectinate setae; outer margin with five or six spine-like setae; ventral surface with fine, simple, scattered setae. Paraglossa sub-rectangular, curved inward; apex rounded; with three rows of long, robust, distally pectinate setae in apical area, sometimes one short, simple seta in anteromedial area, and one short, simple seta in posteromedial area; dorsally with a row of three long, spine-like setae near inner margin. Labial palp with segment I approx. as long as segments II and III combined. Segment I ventrally with short, fine, simple setae. Segment II with short thumb-like, distomedial protuberance; distomedial protuberance 0.3× width of base of segment III; ventral surface with short, simple setae; dorsally with one spine-like seta near outer margin. Segment III broad, rounded; length 0.7× width; ventrally covered with short, spine-like, simple setae and short, fine, simple setae.

***Hind protoptera*** absent.

***Foreleg*** (Fig. 16a, b). Ratio of foreleg segments 1.2:1.0:0.5:0.2. ***Femur*.** Length ca. 3× maximum width. Dorsal margin with a dense row of more than 70 long, curved, spine-like setae and distally some additional long, spine-like setae near margin; length of setae 0.40× maximum width of femur. Apex rounded, with some short, stout setae. Many stout, lanceolate setae scattered along ventral margin; femoral patch absent. ***Tibia*.** Dorsal margin with a dense row of long, spine-like setae. Ventral margin with a row of short, curved, spine-like setae. Anterior surface scattered with short, stout, lanceolate setae. Patellotibial suture present on basal 1/2 area. ***Tarsus*.** Dorsal margin with a row of short, spine-like setae. Ventral margin with a row of short, curved, spine-like setae, distalmost seta much longer. Claw with one row of ten or eleven denticles; distally pointed; with two or three stripes; subapical setae absent.

***Middle and hind legs*** (Fig. 16c, d). As foreleg, but with reduced femoral patch.

***Terga*** (Fig. 16e). Surface with scattered scales and micropores. Posterior margin of tergum IV with discontinuous row of triangular spines, spines wider than long. Triangular spines present on segments IV–VII, absent on segments I–III.

***Gills*** (Fig. 16f). Present on segments II–VII. Margin with small denticles intercalating fine simple setae. Tracheae partly extending from main trunk to inner and outer margins. Gill IV as long as length of segments V and 1/2 VI combined. Gill VII slightly longer than length of segment VIII.

***Paraproct*** (Fig. 16g). Distally not expanded, with 8–12 stout, marginal spines, partly with split tips. Surface scattered with scales and micropores. Cercotractor with numerous small, marginal spines, partly with split tips.

##### Etymology.

Dedicated to the indigenous Onim people of Papua Barat, where the type locality is located.

##### Distribution.

Indonesia: Papua Barat (Fig. 21c).

##### Biological aspects.

The specimens were collected at an altitude of 1200 m, together with *L.arfak* sp. nov.

## Discussion

### Assignment to *Labiobaetis*

For the assignment of the new species to *Labiobaetis* we refer to Kluge and Novikova (2014), Müller-Liebenau (1984b), and McCafferty and Waltz (1995). *Labiobaetis* is characterised by a number of characters, some of which are not found in other taxa (Kluge and Novikova 2014): antennal scape sometimes with a distolateral process (Kaltenbach et al. 2020: fig. 2h); maxillary palp two segmented with excavation at inner distolateral margin of segment II, excavation may be poorly developed or absent (Fig. 1h; Kaltenbach et al. 2020: fig. 2n–p); labium with paraglossae widened and glossae diminished; labial palp segment II with distomedial protuberance (Figs 1i, 6h, 8h, 10h, 13h, 15h). All these characters vary and may be secondarily lost (Kluge and Novikova 2014). The concept of *Labiobaetis* is also based on additional characters, summarised and discussed in Kaltenbach and Gattolliat (2018, 2019).

**Figure 17.**
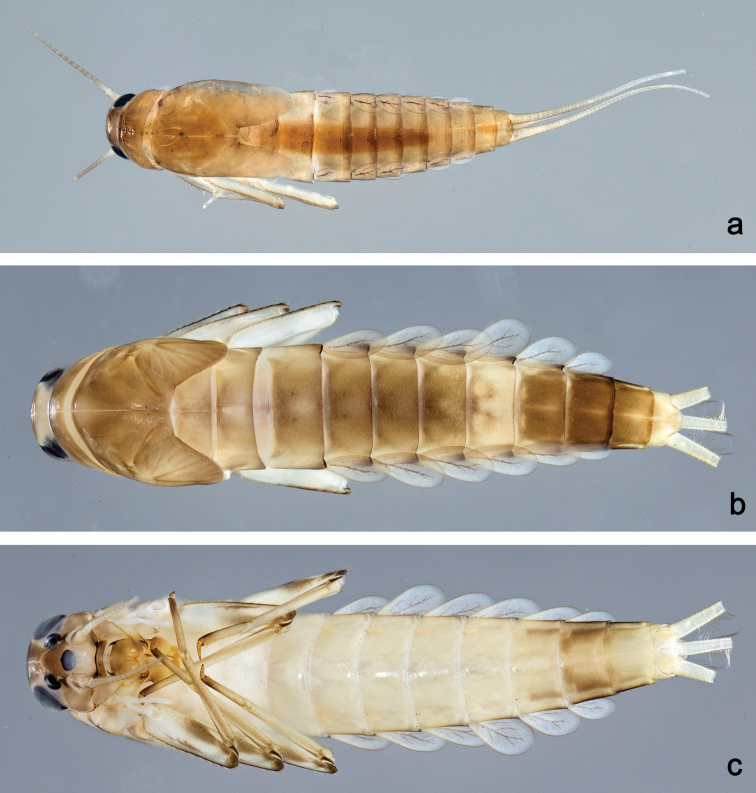
Habitus, larvae: **a***Labiobaetiscatadupa* sp. nov., dorsal view **b***Labiobaetistoraja* sp. nov., dorsal view **c***Labiobaetistoraja* sp. nov., ventral view.

**Figure 18.**
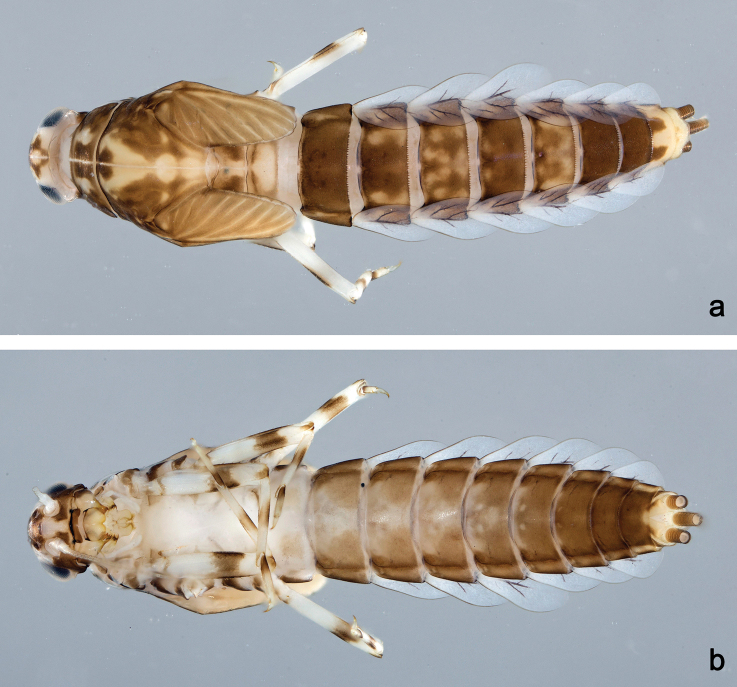
Habitus, larvae: **a***Labiobaetishattam* sp. nov., dorsal view **b***Labiobaetishattam* sp. nov., ventral view.

### *Labiobaetiscatadupa* sp. nov. group

This group is formed for two new species sharing distally very slender glossae, much shorter than paraglossae; an extended thumb-like, hooked protuberance of labial palp segment II; the presence of a long subapical seta on the claw and opposite a rudimentary subapical seta; and the absence of the first pair of gills, hind protoptera and a distolateral process at scape (Figs 1i, 2f, g, 4, 6h, 7h, i). Also, both have a submarginal arc of setae dorsally on the labrum composed of feathered setae. However, the type of these setae of *L.toraja* sp. nov. (Fig. 6a) is common in *Labiobaetis*, present in all species of the groups *operosus* and *difficilis* from Southeast Asia and in almost all species of the Afrotropical realm (Lugo-Ortiz and McCafferty 1997; Gattolliat 2001; Gattolliat et al. 2018; Kaltenbach and Gattolliat 2021a, b). On the contrary, the type of *L.catadupa* sp. nov. with a broadened middle part (Figs 1a, b, 3a, b) is unusual and only known from *L.elouardi* (Gillies, 1993) in West Africa (Kaltenbach and Gattolliat 2021b: fig. 8h–j). Many other characters are very different in *L.elouardi* and *L.catadupa* sp. nov. (see Figs 1, 2; Kaltenbach and Gattolliat 2021b: fig. 8), we therefore assume that these setae evolved independently in both species. The presence of subapical setae on the claw is a first report for *Labiobaetis*. However, one single, long subapical seta is known from other Baetidae (e.g., *Baetodes* Needham & Murphy, 1924; *Gratia* Thomas, 1992, *Indobaetis* Müller-Liebenau & Morihara, 1982) and one on each side was described from e.g., *Baetis* Leach, 1815; *Madaechinopus* Gattolliat & Jacobus, 2010; *Offadens* Lugo-Ortiz & McCafferty, 1998; *Liebebiella* Waltz & McCafferty, 1987, and *Monocentroptilum* Kluge, 2018 (Müller-Liebenau and Morihara 1982; Thomas 1992; Lugo-Ortiz and McCafferty 1999; Gattolliat 2002; Dominguez et al. 2006; Kluge 2018; Yanai et al. 2018).

**Figure 19.**
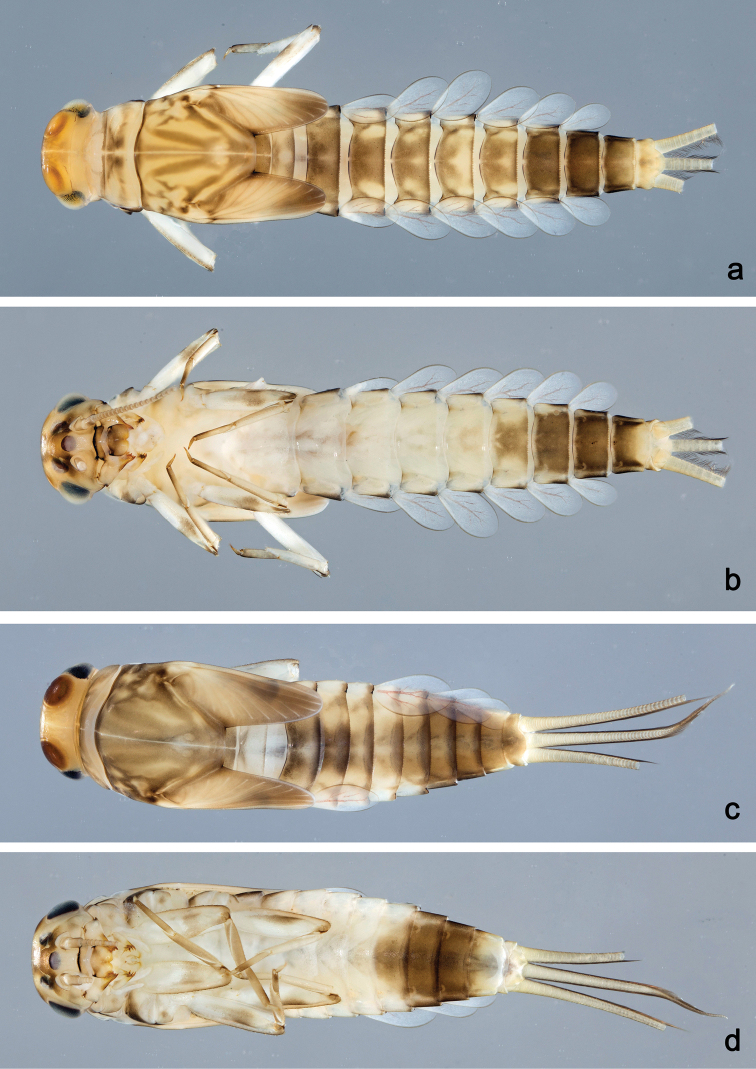
Habitus, larvae: **a***Labiobaetiswerneri* sp. nov., dorsal view **b***Labiobaetiswerneri* sp. nov., ventral view **c***Labiobaetisdendrisetis*, dorsal view **d***Labiobaetisdendrisetis*, ventral view.

**Figure 20.**
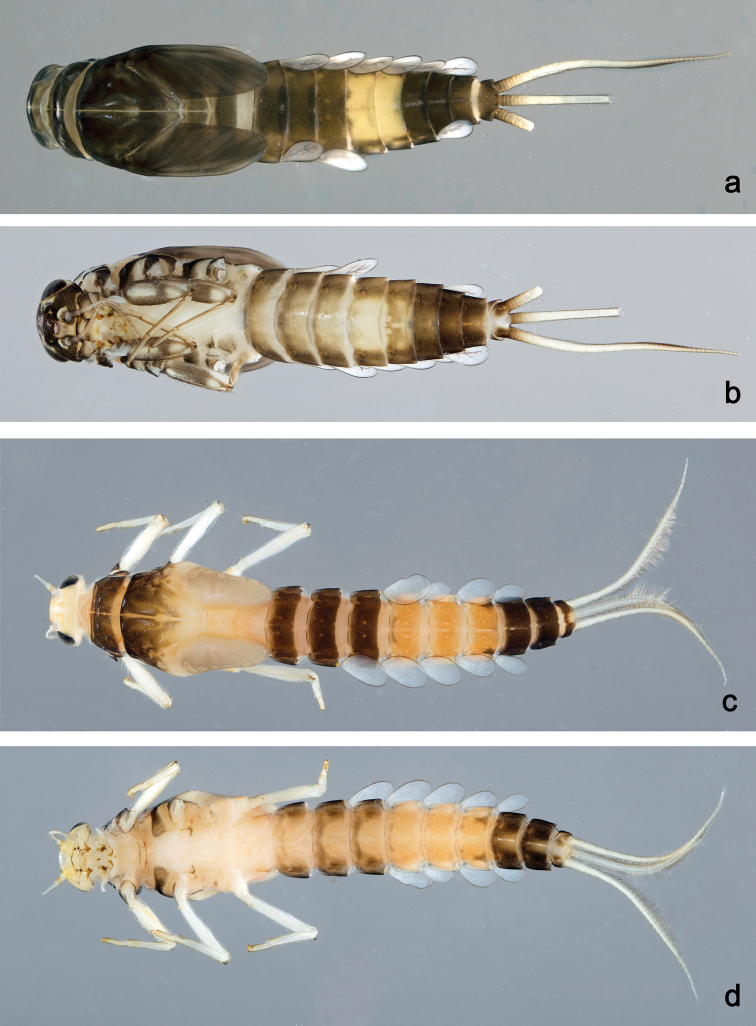
Habitus, larvae: **a***Labiobaetisarfak* sp. nov., dorsal view **b***Labiobaetisarfak* sp. nov., ventral view **c***Labiobaetisonim* sp. nov., dorsal view **d***Labiobaetisonim* sp. nov., ventral view.

The genetic distance (COI, Kimura 2-parameter) between *L.catadupa* sp. nov. and *L.toraja* sp. nov. is 23% and thus well in line with distances found between different species of *Labiobaetis* in Southeast Asia (15%–27% in the Philippines, Kaltenbach et al. 2020; 11%–24% in Indonesia, Kaltenbach and Gattolliat 2019; 19%–25% in Borneo, Kaltenbach and Gattolliat 2020).

### *Labiobaetisclaudiae* group

Based on the discovery of two further species of this group, *L.hattam* sp. nov. and *L.werneri* sp. nov., we adapted the diagnosis of the group (see in the results section) and added another species, *L.centralensis*, already described earlier (Kaltenbach and Gattolliat 2018): *L.hattam* sp. nov. and *L.centralensis* have only short setae at the gills margin and not alternately shorter and longer setae, as it is the case in the other species. However, all other diagnostic characters of the *claudiae* group are present in both species as well and especially the presence of a femoral patch on all legs, which is rare in *Labiobaetis* in this region, is considered to be a strong character. Therefore, we include these two species in this group as well.

**Figure 21.**
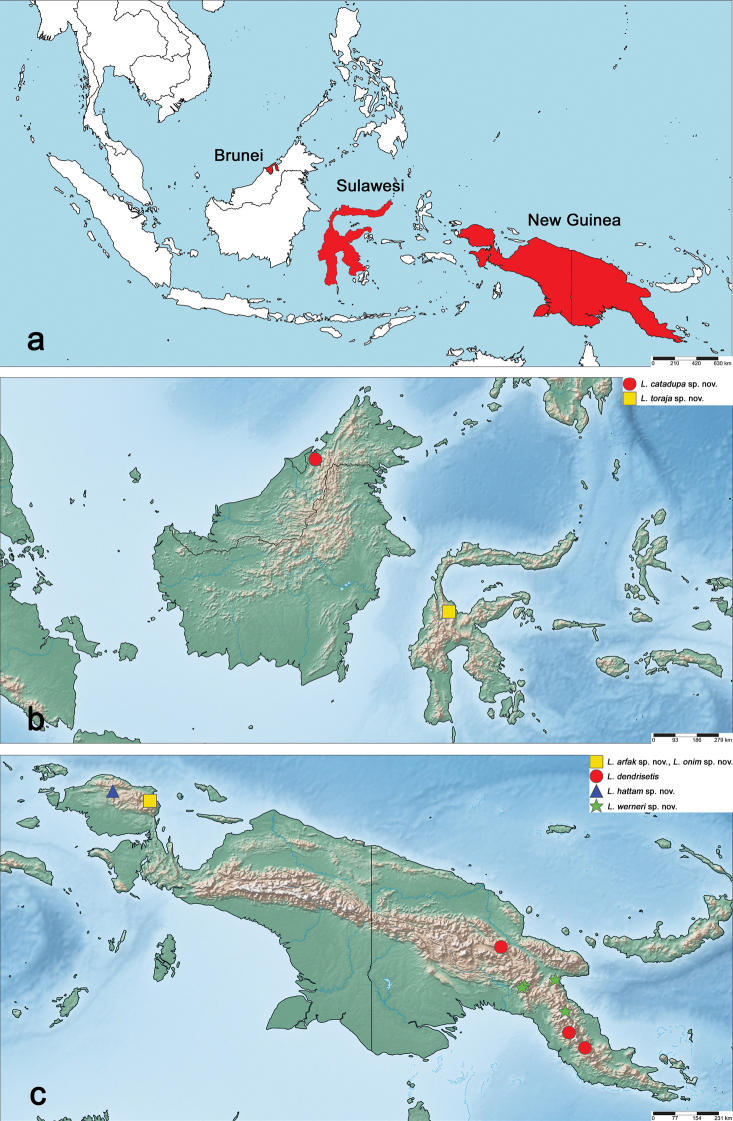
Maps: **a** overview of the region treated in this study (marked in red) **b** distribution of the new *Labiobaetis* species in Borneo and Sulawesi **c** distribution of the treated *Labiobaetis* species in New Guinea.

**Table 2.** GPS coordinates of locations of examined specimens.

Species	Locality	GPS coordinates
*L.catadupa* sp. nov.	Brunei: Temburong National Park	04°32'49"N, 115°09'30"E
		04°33'10"N, 115°09'20"E
		04°32'42"N, 115°09'31"E
		04°32'23"N, 115°09'34"E
		04°32'51"N, 115°09'25"E
		04°32'56"N, 115°09'27"E
		04°33'39"N, 115°08'51"E
		04°33'39"N, 115°08'54"E
*L.toraja* sp. nov.	Sulawesi	01°19'35"S, 120°18'40"E
*L.hattam* sp. nov.	Papua Barat	00°52'29"S, 132°46'06"E
*L.werneri* sp. nov.	Papua New Guinea	07°01'42"S, 145°49'48"E
	Papua New Guinea	07°05'40"S, 145°44'28"E
	Papua New Guinea	06°51'04"S, 146°48'04"E
	Papua New Guinea	07°51'02"S, 147°07'00"E
*L.dendrisetis*	Papua New Guinea: Simbu Prov.	05°49' 00"S, 145°04'30"E
	Papua New Guinea: Central Prov.	08°31'35"S, 147°14'06"E
	Papua New Guinea: Central Prov.	08°31'17"S, 147°13'41"E
	Papua New Guinea: Central Prov.	09°00'20"S, 147°44'15"E
*L.arfak* sp. nov.	Papua Barat	01°10'59"S, 133°56'51"E
*L.onim* sp. nov.	Papua Barat	01°10'59"S, 133°56'51"E

The interspecific genetic distances between the species of the *claudiae* group are rather high, between 18% and 27% (Table 3), which is in line with the values reported for other *Labiobaetis* species in New Guinea (13%–28%; Kaltenbach and Gattolliat 2018).

**Figure 22.**
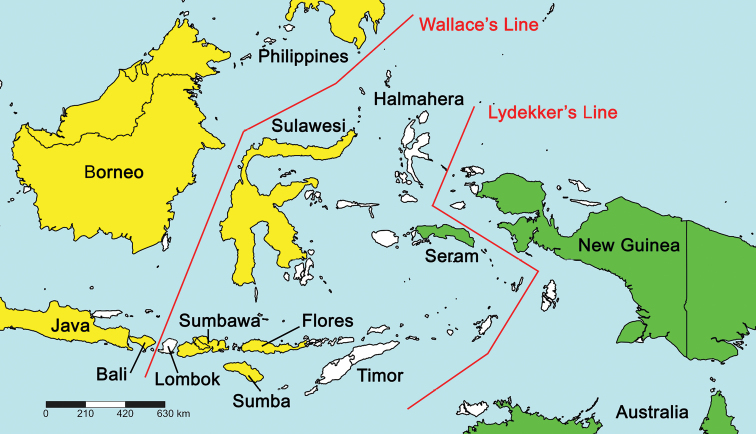
Distribution of *Labiobaetis* in the Wallacea, with indication of morphological affinities to species of the Oriental realm (yellow) or New Guinea (green). White: Islands without reported species. Wallace’s Line and Lydekker’s Line adapted after Cox et al. 2016: fig. 11.9.

**Table 3.** Intraspecific (bold) and interspecific genetic distances of the species of the *L.claudiae* group (COI; Kimura 2-parameter).

		1	2	3	4	5	6	7	8	9
**1**	*L.academicus*									
**2**	*L.academicus*	**0.00**								
**3**	*L.centralensis*	0.22	0.22							
**4**	*L.centralensis*	0.23	0.23	**0.01**						
**5**	*L.claudiae*	0.21	0.21	0.24	0.24					
**6**	*L.hattam* sp. nov.	0.23	0.23	0.23	0.23	0.23				
**7**	*L.stagnum*	0.20	0.20	0.26	0.27	0.23	0.23			
**8**	*L.werneri* sp. nov.	0.19	0.19	0.25	0.25	0.20	0.21	0.23		
**9**	*L.werneri* sp. nov.	0.18	0.18	0.25	0.25	0.20	0.21	0.22	**0.03**	
**10**	*L.werneri* sp. nov.	0.19	0.19	0.25	0.25	0.20	0.21	0.22	0.05	**0.06**

Ball et al. (2005) reported a mean interspecific, congeneric distance of 18% for mayflies from the United States and Canada. The intraspecific distances are very low in most cases as expected, ranging from 0% to 3% (K2P). This result is certainly biased as it is based on a limited number of sequenced specimens per species, which were partly from a single population. The exception is *L.werneri* sp. nov., where one of the three sequenced specimen has a distance of 5% and 6% respectively to the two other sequenced specimens. Here, the larger genetic distance may be explained by a possible isolation of some locations in the central mountain chain of New Guinea, where the species occurs (Fig. 21c). Intraspecific distances of 4%–6% were also reported in some cases for other *Labiobaetis* species in New Guinea, Indonesia, Borneo, and the Philippines (Kaltenbach and Gattolliat 2018, 2019, 2020; Kaltenbach et al. 2020), as well as in aquatic beetles in the Philippines (Komarek and Freitag 2020). Ball et al. (2005) also reported a case with 6% intraspecific distance in a mayfly in North America and intraspecific K2P distances of more than 3.5% are not uncommon within Plecoptera as well (Gill et al. 2015; Gattolliat et al. 2016).

### *Labiobaetisseramensis* group

Due to the discovery of two new species of the *L.seramensis* group in New Guinea, *L.arfak* sp. nov. and *L.onim* sp. nov., and the re-examination of the types of *L.seramensis* and *L.wahai* from Seram (Indonesia), we could complement the diagnosis of this group (see in section results). The presence of a reduced femoral patch on middle and hind legs and its absence on forelegs is identified as an additional character of the group. As femoral patches are generally rare in *Labiobaetis* in Southeast Asia and New Guinea, it is considered to be a strong character.

*Labiobaetisonim* sp. nov. has three remarkable characters, which are atypical for *Labiobaetis*: the femur has a very dense and long setation with more than 70 setae at the dorsal margin, the tibia also has a dense and rather long setation along its dorsal margin and the posterior margins of the tergites have a discontinuous row of triangular spines, similar as in *Baetisnoa* Yanai & Gattolliat, 2018 from Israel (Yanai et al. 2018: fig. 13C). However, most characters of the *L.seramensis* group are present in *L.onim* sp. nov. and the mouthparts are generally very similar to *L.arfak* sp. nov. On the other hand, a femoral patch is absent on the foreleg, contrary to what should be expected for *Baetis* and the labial palp has the characteristics of *Labiobaetis*. We could not investigate the folding of the protogonostyli developing under the larval cuticle of last instar male larvae. We are convinced that the remarkable posterior margins of the tergites are a convergent development and that the species belongs to the *seramensis* group of *Labiobaetis*.

## Distribution of *Labiobaetis* in the Wallacea region

The *L.seramensis* group is present with two species on the island Seram (Indonesia, Moluccas) and with two others in New Guinea. Moreover, *L.arfak* sp. nov. from New Guinea is morphologically very similar to *L.seramensis* from Seram (Kaltenbach and Gattolliat 2018: figs. 38, 39). Differences are the dorsal setation of tibia and tarsus and two posterolateral spines on tergites VIII and IX in *L.arfak* sp. nov. (Fig. 14f), absent in *L.seramensis*. Kaltenbach and Gattolliat (2018) already discussed the general morphological affinities of *L.seramensis* and *L.wahai* with species from New Guinea rather than with species from the Oriental realm. *Labiobaetis* from New Guinea (and Australia) are characterised by the absence of an antennal scape process, all but one species have only six pairs of gills, hind protoptera are absent in all species, and most species have simple setae forming the submarginal arc of setae on the dorsal surface of the labrum. The number of setae at the dorsal margin of the femur is mostly above 20, sometimes even above 40, and only in one case less than 12 (Kaltenbach and Gattolliat 2018). In the Oriental realm as well as in other regions, these character states are more evenly distributed and there are at least several species with or without antennal scape process, with six or seven pairs of gills, and with or without hind protoptera. The proportion of the different types of dorsal labrum arc setae (simple, feathered, clavate) is more equalised. The latter is especially true in the Oriental realm, whereas only the feathered type is present in the Afrotropical region (Lugo-Ortiz and McCafferty 1997; Gattolliat 2001; Kaltenbach and Gattolliat 2019, 2020, 2021a, b; Kaltenbach et al. 2020). Additionally, the number of setae at the dorsal margin of the femur in the Oriental realm is usually below 20 and often below 12.

The Wallace Line is marking the eastern boundary of the Oriental fauna, and Lydekker’s Line is considered to be the western boundary of the strictly Australian fauna. The mixed zone in between is referred to as Wallacea by many biogeographers (Cox et al. 2016: fig. 11.9). It encompasses Sulawesi, Halmahera, the Moluccas, the Lesser Sunda Islands (e.g., Lombok, Sumbawa, Sumba, Flores, Timor) and many smaller islands. For *Labiobaetis*, the two reported species of Seram are clearly faunal elements of New Guinea, but all other known species of the Wallacea have strong morphological affinities to the Oriental realm (Fig. 22): *L.pilosus* Kaltenbach & Gattolliat, 2019 (Sulawesi) is part of the *numeratus* group, which is widely distributed in the Oriental realm, but absent in New Guinea; *L.itineris* Kaltenbach & Gattolliat, 2019 (Bali, Sumbawa) is part of the *sumigarensis* group, which is widely distributed in the Oriental realm, but absent in New Guinea; *L.weifangae* Kaltenbach & Gattolliat, 2019 (Sumbawa, Sumba), L.cf.weifangae (unpublished, Flores) and *L.jonasi* Kaltenbach & Gattolliat, 2019 (Sumba) are part of or very close (for *L.jonasi*) to the *difficilis* group, distributed in Southeast Asia, but absent in New Guinea; *L.sulawesiensis* Kaltenbach & Gattolliat, 2019 (Sulawesi) and *L.sumbensis* Kaltenbach & Gattolliat, 2019 (Sumba) belong to the *batakorum* group, additionally present in Sumatra and absent in New Guinea.

Independent from the situation in the Wallacea, there could have been a limited stepping stone exchange between the Philippines and New Guinea, as we found members of the groups *vallus* and *dendrisetis* in both these archipelagos (Kaltenbach and Gattolliat 2018; Kaltenbach et al. 2020) and both groups are unknown from other areas.

Taking into account the extreme diversity in Southeast Asia and New Guinea, the rather poor collection activities in the past, with many still unexplored regions, and the obvious richness of *Labiobaetis* in this region, we have to expect many more species with further collections in the future.

## Supplementary Material

XML Treatment for Labiobaetis catadupa

XML Treatment for Labiobaetis toraja

XML Treatment for Labiobaetis hattam

XML Treatment for Labiobaetis werneri

XML Treatment for Labiobaetis dendrisetis

XML Treatment for Labiobaetis arfak

XML Treatment for Labiobaetis onim

## References

[B1] Allison A (2010) New Guinea, Biology. In: Gillespie RG, Clague DA (Eds) Encyclopedia of islands, University of California Press, Berkeley, Los Angeles, London, 652–659.

[B2] Baker K Chadwick M Sulaiman ZH (2016a) Eco-hydromorphic classification for understanding stream macroinvertebrate biodiversity in Brunei Darussalam, Northern Borneo. Zoological Studies 55: 1–27. 10.6620/ZS.2016.55-37 PMC6511898 31966182

[B3] Baker K Chadwick M Kahar RS Sulaiman ZH Wahab RHA (2016b) Fluvial biotopes influence macroinvertebrate biodiversity in South-East Asian tropical streams. Ecosphere 7: 1–15.

[B4] Baker K Chadwick M Wahab RAH Kahar RS (2017a) Benthic community structure and ecosystems functions in above- and below-waterfall pools in Borneo. Hydrobiologia 787: 1–16.

[B5] Baker K Chadwick M McGill RAR Wahab RHA Kahar RS (2017b) Macroinvertebrate trophic structure on waterfalls in Borneo. Marine and Freshwater Research 68: 2061–2069.

[B6] Baker K Damken C Gattolliat J-L Grafe U Kahar R Orr A Sartori M Wahab RA Zettel H Chadwick MA (2019) Carpooling with ecologists, geographers and taxonomists: perceptions from conducting environmental research in tropical regions. Biodiversity and Conservation 28: 957–981. https://doi.org/10.1007/s10531-018-01695-3

[B7] Ball SL Hebert PDN Burian SK Webb JM (2005) Biological identifications of mayflies (Ephemeroptera) using DNA barcodes. Journal of the North American Benthological Society 24: 508–524. https://doi.org/10.1899/04-142.1

[B8] Barber-James HM Sartori M Gattolliat J-L Webb J (2013) World checklist of freshwater Ephemeroptera species. http://fada.biodiversity.be/group/show/35

[B9] Chakrabarty P Warren M Page LM Baldwin CC (2013) GenSeq: An updated nomenclature and ranking for genetic sequences from type and non-type sources. ZooKeys 346: 29–41. https://doi.org/10.3897/zookeys.346.5753 10.3897/zookeys.346.5753 PMC3821064 24223486

[B10] Cox CB Moore PD Ladle RJ (2016) Biogeography. John Whiley & Sons, Ltd, West Sussex, 482 pp.

[B11] Cruz PV Nieto C Gattolliat J-L Salles FF Hamada N (2020) A cladistic insight into higher level classification of Baetidae (Insecta: Ephemeroptera). Systematic Entomology 2020: 12 pp.

[B12] Dominguez E Molineri C Pescador ML Hubbard MD Nieto C (2006) Ephemeroptera of South America. In: Adis J, Arias JR, Rueda-Delgado G, Wantzen KM (Eds) Aquatic Biodiversity in Latin America, Vol. 2. Pensoft Publishers, Sofia-Moscow, 646 pp.

[B13] Folmer O Black M Hoeh W Lutz R Vrijenhoek R (1994) DNA primers for amplification of mitochondrial cytochrome c oxidase subunit I from divers metazoan invertebrates. Molecular Marine Biology and Biotechnology 3: 294–299. http://www.mbari.org/staff/vrijen/PDFS/Folmer_94MMBB.pdf 7881515

[B14] Gattolliat J-L (2001) Six new species of *Labiobaetis* Novikova & Kluge (Ephemeroptera: Baetidae) from Madagascar with comments on the validity of the genus. Annales de Limnologie 37: 97–123. https://doi.org/10.1051/limn/2001013

[B15] Gattolliat J-L (2002) Two new genera of Baetidae (Ephemeroptera; Insecta) from Madagascar. Aquatic Insects 24: 143–159. https://doi.org/10.1076/aqin.24.2.143.4903

[B16] Gattolliat J-L Kondratieff BC Kaltenbach T Al Dhafer HM (2018) *Labiobaetis* from the Kingdom of Saudi Arabia (Insecta: Ephemeroptera: Baetidae). ZooKeys 774: 77–104. https://doi.org/10.3897/zookeys.774.25273 10.3897/zookeys.774.25273 PMC6056567 30057465

[B17] Gattolliat J-L Nieto C (2009) The family Baetidae (Insecta: Ephemeroptera): synthesis and future challenges. Aquatic Insects 31: 41–62. https://doi.org/10.1080/01650420902812214

[B18] Gattolliat J-L Vinçon G Wyler S Pawlowski J Sartori M (2016) Toward a comprehensive COI DNA barcode library for Swiss Stoneflies (Insecta: Plecoptera) with special emphasis on the genus *Leuctra*. Zoosymposia 11: 135–155. https://doi.org/10.11646/zoosymposia.11.1.15

[B19] Gill BA Sandberg JB Kondratieff BC (2015) Evaluation of the morphological species concepts of 16 western Nearctic *Isoperla* species (Plecoptera: Perlodidae) and their respective species groups using DNA barcoding. Illiesia 11: 130–146. http://illiesia.speciesfile.org/papers/Illiesia11-11.pdf

[B20] Hall R (2010) Indonesia, Geology. In: Gillespie RG Clague DA (Eds) Encyclopedia of islands.University of California Press, Berkeley, Los Angeles, London, 454–460.

[B21] Hubbard MD (1995) Towards a standard methodology for the description of mayflies (Ephemeroptera). In: Corkum LD Ciborowski JJH (Eds) Current directions in research on Ephemeroptera.Canadian Scholar’s Press, Toronto, 361–369.

[B22] Jacobus LM Macadam CR Sartori M (2019) Mayflies (Ephemeroptera) and their contributions to ecosystem services. Insects 10: 1–26. https://doi.org/10.3390/insects10060170 10.3390/insects10060170 PMC6628430 31207933

[B23] Kaltenbach T Gattolliat J-L (2018) The incredible diversity of *Labiobaetis* Novikova & Kluge in New Guinea revealed by integrative taxonomy (Ephemeroptera, Baetidae). ZooKeys 804: 1–136. https://doi.org/10.3897/zookeys.804.28988 10.3897/zookeys.804.28988 PMC6297208 30584389

[B24] Kaltenbach T Gattolliat J-L (2019) The tremendous diversity of *Labiobaetis* Novikova & Kluge in Indonesia (Ephemeroptera, Baetidae). ZooKeys 895: 1–117. https://doi.org/10.3897/zookeys.895.38576 31844411 10.3897/zookeys.895.38576 PMC6906171

[B25] Kaltenbach T Gattolliat J-L (2020) *Labiobaetis* Novikova & Kluge in Borneo (Ephemeroptera, Baetidae). ZooKeys 914: 43–79. https://doi.org/10.3897/zookeys.914.47067 32132855 10.3897/zookeys.914.47067 PMC7046705

[B26] Kaltenbach T Gattolliat J-L (2021a) *Labiobaetis* Novikova & Kluge in Ethiopia (Ephemeroptera, Baetidae), with description of a new species. African Invertebrates 62: 231–255.

[B27] Kaltenbach T Gattolliat J-L (2021b) *Labiobaetis* Novikova & Kluge in West Africa (Ephemeroptera, Baetidae), with description of a new species. African Invertebrates 62: 355–382.

[B28] Kaltenbach T Garces JM Gattolliat J-L (2020) The success story of *Labiobaetis* Novikova & Kluge in the Philippines (Ephemeroptera, Baetidae), with description of 18 new species. ZooKeys 1002: 1–114. https://doi.org/10.3897/zookeys.1002.58017 33363429 10.3897/zookeys.1002.58017 PMC7746671

[B29] Kaltenbach T Surbakti S Kluge NJ Gattolliat J-L Sartori M Balke M (2021) Discovery of a new mayfly species near (Ephemeroptera, Baetidae) Cenderawasih University campus in Papua, Indonesia. Treubia 48: 37–54.

[B30] Kimura M (1980) A simple method for estimating evolutionary rates of base substitutions through comparative studies of nucleotide sequences. Journal of Molecular Evolution 16: 111–120. https://doi.org/10.1007/BF01731581 7463489 10.1007/BF01731581

[B31] Kingston T (2010) Indonesia, Biology. In: Gillespie RG Clague DA (Eds) Encyclopedia of islands.University of California Press, Berkeley, Los Angeles, London, 446–453.

[B32] Kluge NJ (2004) The phylogenetic system of Ephemeroptera. Academic Publishers, Dordrecht, 442 pp. https://doi.org/10.1007/978-94-007-0872-3

[B33] Kluge NJ (2018) A new Afrotropical genus *Monocentroptilum* gen. nov. (Ephemeroptera: Baetidae: Protopatellata). Zootaxa 4486: 115–128. https://doi.org/10.11646/zootaxa.4486.2.2 10.11646/zootaxa.4486.2.2 30313755

[B34] Kluge NJ Novikova EA (2014) Systematics of *Indobaetis* Müller-Liebenau & Morihara 1982, and related implications for some other Baetidae genera (Ephemeroptera). Zootaxa 3835: 209–236. https://doi.org/10.11646/zootaxa.3835.2.3 25081445 10.11646/zootaxa.3835.2.3

[B35] Kluge NJ Novikova EA (2016) New tribe Labiobaetini tribus n., redefinition of *Pseudopannota* Waltz & McCafferty 1987 and descriptions of new and little known species from Zambia and Uganda. Zootaxa 4169: 1–43. https://doi.org/10.11646/zootaxa.4169.1.1 27701309 10.11646/zootaxa.4169.1.1

[B36] Komarek A Freitag H (2020) Taxonomic revision of *Agraphydrus* Régimbart, 1903. IV. Philippines. (Coleoptera: Hydrophilidae: Acidocerinae). Koleopterologische Rundschau 90: 201–242.

[B37] Kumar S Stecher G Tamura K (2016) MEGA 7: molecular evolutionary genetics analysis version 7.0 for bigger data sets. Molecular Biology and Evolution 33: 1870–1874. https://doi.org/10.1093/molbev/msw054 27004904 10.1093/molbev/msw054 PMC8210823

[B38] Lugo-Ortiz CR McCafferty WP (1997) *Labiobaetis* Novikova & Kluge (Ephemeroptera: Baetidae) from the Afrotropical region. African Entomology 5: 241–260.

[B39] Lugo-Ortiz CR McCafferty WP (1999) *Edmundsiopsinstigatus*: a new genus and species of small minnow mayflies (Ephemeroptera: Baetidae) from Australia. Entomological News 110: 65–69.

[B40] Lugo-Ortiz CR McCafferty WP Waltz RD (1999) Definition and reorganization of the genus *Pseudocloeon* (Ephemeroptera: Baetidae) with new species descriptions and combinations. Transactions of the American Entomological Society 125: 1–37.

[B41] McCafferty WP Waltz RD (1995) *Labiobaetis* (Ephemeroptera: Baetidae): new status, new North American species, and related new genus. Entomological News 106: 19–28.

[B42] Müller-Liebenau I (1981) Review of the original materiel of the baetid genera *Baetis* and *Pseudocloeon* from the Sunda Islands and the Philippines described by G. Ulmer, with some general remarks (Insecta: Ephemeroptera). Mitteilungen aus dem hamburgischen Zoologischen Museum und Institut 78: 197–208.

[B43] Müller-Liebenau I (1984a) Baetidae from Sabah (East Malaysia) (Ephemeroptera). In: Landa V, Soldán T, Tonner M (Eds) Proceedings of the Fourth International Conference on Ephemeroptera, Czechoslovak Academy of Sciences, Budejovice, 85–89.

[B44] Müller-Liebenau I (1984b) New genera and species of the family Baetidae from West-Malaysia (River Gombak) (Insecta: Ephemeroptera). Spixiana 7: 253–284.

[B45] Müller-Liebenau I Morihara DK (1982) *Indobaetis*: a new genus of Baetidae from Sri Lanka (Insecta: Ephemeroptera) with two new species. Gewässer und Abwässer 68/69: 26–34.

[B46] Novikova EA Kluge NJ (1987) Systematics of the genus *Baetis* (Ephemeroptera, Baetidae), with descriptions of new species from Middle Asia. Vestnik Zoologii 1987(4): 8–19. [in Russian]

[B47] Odgen TH Whiting MF (2005) Phylogeny of Ephemeroptera (mayflies) based on molecular evidence. Molecular Phylogenetics and Evolution 37: 625–643. https://doi.org/10.1016/j.ympev.2005.08.008 16214375 10.1016/j.ympev.2005.08.008

[B48] Ogden TH Breinholt JW Bybee SM Miller DB Sartori M Shiozawa D Whiting MF (2019) Mayfly phylogenomics: initial evaluation of anchored hybrid enrichment data for the order Ephemeroptera. Zoosymposia 16: 167–181.

[B49] Ogden TH Gattolliat J-L Sartori M Staniczek AH Soldan T Whiting MF (2009) Towards a new paradigm in mayfly phylogeny (Ephemeroptera): combined analysis of morphological and molecular data. Systematic Entomology 34: 616–634. https://doi.org/10.1111/j.1365-3113.2009.00488.x

[B50] Quek S-P (2010) Borneo. In: Gillespie RG Clague DA (Eds) Encyclopedia of islands.University of California Press, Berkeley, Los Angeles, London, 111–116.

[B51] Sanger F Nicklen S Coulson AR (1977) DNA sequencing with chain-terminating inhibitors. Proceedings of the National Academy of Sciences USA 74: 5463–5467. 10.1073/pnas.74.12.5463 PMC431765 271968

[B52] Sartori M Brittain JE (2015) Order Ephemeroptera. In: Thorp J Rogers DC (Eds) Ecology and general biology: Thorp and Corvich’s Freshwater Invertebrates.Academic Press, 873–891. https://doi.org/10.1016/B978-0-12-385026-3.00034-6

[B53] Shi W Tong X (2014) The genus *Labiobaetis* (Ephemeroptera: Baetidae) in China, with description of a new species. Zootaxa 3815: 397–408. https://doi.org/10.11646/zootaxa.3815.3.5 10.11646/zootaxa.3815.3.5 24943622

[B54] Shorthouse DP (2010) SimpleMappr, an online tool to produce publication-quality point maps. https://www.simplemappr.net [Accessed March 03, 2021]

[B55] Thomas A (1992) *Gratiasororculaenadinae* n. gen., n. sp., Ephéméroptère nouveau de Thailande (Ephemeroptera, Baetidae). Bulletin de la Société d’Histoire Naturelle de Toulouse 128: 47–51.

[B56] Tofilski A (2018) DKey software for editing and browsing dichotomous keys. ZooKeys 735: 131–140. https://doi.org/10.3897/zookeys.735.21412 10.3897/zookeys.735.21412 PMC5904324 29674865

[B57] Toussaint EFA Sagata K Surbakti S Hendrich L Balke M (2013) Australasian sky islands act as a diversity pump facilitating peripheral speciation and complex reversal from narrow endemic to widespread ecological supertramp. Ecology and Evolution 3: 1031–1049. https://doi.org/10.1002/ece3.517 23610642 10.1002/ece3.517 PMC3631412

[B58] Toussaint E.A Hall R Monaghan MT Sagata K Ibalim S Shaverdo HV Vogler AP Pons J Balke M (2014) The towering orogeny of New Guinea as a trigger for arthropod megadiversity. Nature Communications 5: 4001–4010. https://doi.org/10.1038/ncomms5001 10.1038/ncomms5001 24874774

[B59] Ulmer G (1913) Ephemeriden aus Java, gesammelt von Edw. Jacobsen. Notes from the Leiden Museum 35: 102–120.

[B60] Ulmer G (1924) Ephemeropteren von den Sunsa-Inseln und den Philippinen. Treubia 6: 28–91.

[B61] Ulmer G (1939) Eintagsfliegen (Ephemeropteren) von den Sunda-Inseln. Archiv für Hydrobiologie, Supplement 16: 443–692.

[B62] Vuataz L Sartori M Wagner A Monaghan MT (2011) Toward a DNA taxonomy of Alpine *Rhithrogena* (Ephemeroptera: Heptagenidae) using a mixed Yule-Coalescent Analysis of mitochondrial and nuclear DNA. PLoS ONE 6: 1–11. https://doi.org/10.1371/journal.pone.0019728 10.1371/journal.pone.0019728 PMC3096624 21611178

[B63] Yanai Z Gattolliat J-L Dorchin N (2018) Taxonomy of *Baetis* Leach in Israel (Ephemeroptera, Baetidae). ZooKeys 794: 45–84. https://doi.org/10.3897/zookeys.794.28214 10.3897/zookeys.794.28214 PMC6224371 30416340

